# Computer-Based Simulation Technologies in Pediatric Cardiovascular Diseases: A Systematic Review of Applications and Outcomes

**DOI:** 10.3390/healthcare14183086

**Published:** 2026-09-19

**Authors:** Arezoo Abasi, Haleh Ayatollahi, Amirhossein Amirzadeh

**Affiliations:** 1 Student Research Committee, Iran University of Medical Sciences, Tehran 14496-14535, Iran; 2 Health Management and Economics Research Center, Health Management Research Institute, Iran University of Medical Sciences, Tehran 19967-13883, Iran; 3 Department of Health Information Management, School of Health Management and Information Sciences, Iran University of Medical Sciences, Tehran 19967-13883, Iran

**Keywords:** pediatric cardiovascular diseases, computer-based simulation, virtual reality, augmented reality, three-dimensional modeling

## Abstract

**Introduction**: Computer-based simulation technologies, including virtual reality (VR), augmented reality (AR), mixed reality (MR), and three-dimensional (3D) modeling, are increasingly used in pediatric cardiovascular care. Despite growing adoption of these technologies, no comprehensive synthesis across VR, AR, MR, and 3D modeling modalities exists for pediatric cardiovascular care. **Objective**: This review aimed to synthesize evidence regarding the applications and outcomes of computer-based simulation technologies in pediatric cardiovascular diseases. **Methods**: A systematic review was conducted by searching nine databases (PubMed, Web of Science, Scopus, Ovid, the Cochrane Library, IEEE Xplore, ProQuest, CINAHL, and EBSCO Host) for eligible studies published up to 1 September 2025. Thirty-six studies were included and appraised using the Mixed Methods Appraisal Tool (MMAT), the Critical Appraisal Skills Programme (CASP) checklist, and the ROBINS-I tool. Substantial clinical and methodological heterogeneity precluded meta-analysis. **Results:** VR was the most frequently used modality (n = 14), followed by 3D modeling (n = 8), multimodal approaches (n = 8), MR (n = 6), and AR (n = 3). This technology was mainly used for congenital heart defects (n = 18). MR holography improved diagnostic accuracy for complex anomalies (95.5% vs. 89.7%); VR-based surgical plans aligned better with the real surgery than did 2D imaging plans (80% vs. 66%); VR was preferred over 3D-printed models by 87% of participants (8.5/10 vs. 6.3/10 for anatomical understanding); and VR curricula (Stanford Virtual Heart) significantly increased CHD knowledge scores among students and residents (*p* < 0.05). However, this evidence was predominantly derived from small, single-center observational studies, with barriers including hardware limitations and limited long-term outcome data. **Conclusions**: Computer-based simulation technologies show considerable potential for surgical planning, diagnostic assessment, and education in pediatric cardiovascular diseases. They can be used as complementary tools rather than replacements for standard imaging or clinical judgment. While VR and 3D modeling show promise for surgical planning and education, multicenter comparative studies with standardized outcomes and cost-effectiveness analyses are essential before routine clinical implementation.

## 1. Introduction

Cardiovascular diseases are a leading cause of mortality in children worldwide [1]. Among them, congenital heart defects (CHDs) affect 0.8–1.2% of live births and account for a substantial share of pediatric cardiac morbidity [2]. Acquired conditions such as rheumatic heart disease, Kawasaki disease, and myocarditis also contribute to the burden, though CHDs remain predominant in most reports [1,3]. Severity ranges from mild to life-threatening, and treatment involves medication, surgery, or catheter-based intervention [4]. CHDs affect 1.5 million children annually and are a leading cause of infant mortality [5,6,7]; approximately 25% require surgical correction, and the complex three-dimensional anatomy of many lesions poses substantial challenges for diagnosis, preoperative planning, and treatment [5,6,7,8]. Accurate understanding of spatial relationships among cardiac structures is particularly critical for complex lesions, where conventional representations may not fully convey patient-specific anatomy.

Current diagnosis and surgical planning rely on two-dimensional (2D) imaging, computed tomography, magnetic resonance imaging, and echocardiography, which often fail to fully capture the intricate 3D architecture of congenital lesions [6,9]. Reconstructing 3D mental models from 2D images depends heavily on the specialist’s visualization skills, introducing inter-observer variability and limiting training efficiency [5,9]. In addition, pediatric cardiology training programs vary widely in structure and may not provide sufficient repeated exposure to complex three-dimensional anatomy [10].

In response, computer-generated 3D heart models and immersive simulations have emerged as promising tools for education and planning, enabling repetitive, risk-free practice [11]. These include standalone modalities such as virtual reality (VR), augmented reality (AR), mixed reality (MR), and 3D modeling software, as well as multimodal approaches that combine two or more visualization techniques [12,13], and have been used for patient-specific anatomical visualization, surgical planning, diagnostic support, medical education, and clinical decision-making. Prior evidence suggests benefits of 3D-based technologies for surgical planning and outcomes [14]. Similarly, immersive interactive preoperative assessment via VR [5] and simulation-based training have been identified as an important educational strategy, particularly in pediatric cardiac intensive care units, where patients are vulnerable to rapid deterioration [15,16]. This educational value is supported by established learning theories, including deliberate practice, experiential learning, and cognitive load theory, which facilitate skill acquisition through repetitive practice and structured feedback [17,18].

However, while some meta-analyses have focused on single technologies such as 3D printing, a comprehensive synthesis spanning the full range of computer-based simulation modalities is critically lacking. Moreover, prior reviews have largely assessed individual technologies in isolation and focused on a specific disease or health condition, without comparing the outcomes of different technologies [19]. Despite growing adoption of these technologies, no comprehensive synthesis across VR, AR, MR, and 3D modeling modalities exists for pediatric cardiovascular care.

Because pediatric cardiovascular conditions differ substantially in pathophysiology, clinical management, and simulation objectives, this review aimed to synthesize evidence regarding the applications and outcomes of computer-based simulation technologies in pediatric cardiovascular diseases. The primary research question was as follows:

What are the applications and outcomes of computer-based simulation technologies in pediatric cardiovascular diseases?

## 2. Methods

**Figure 1:**
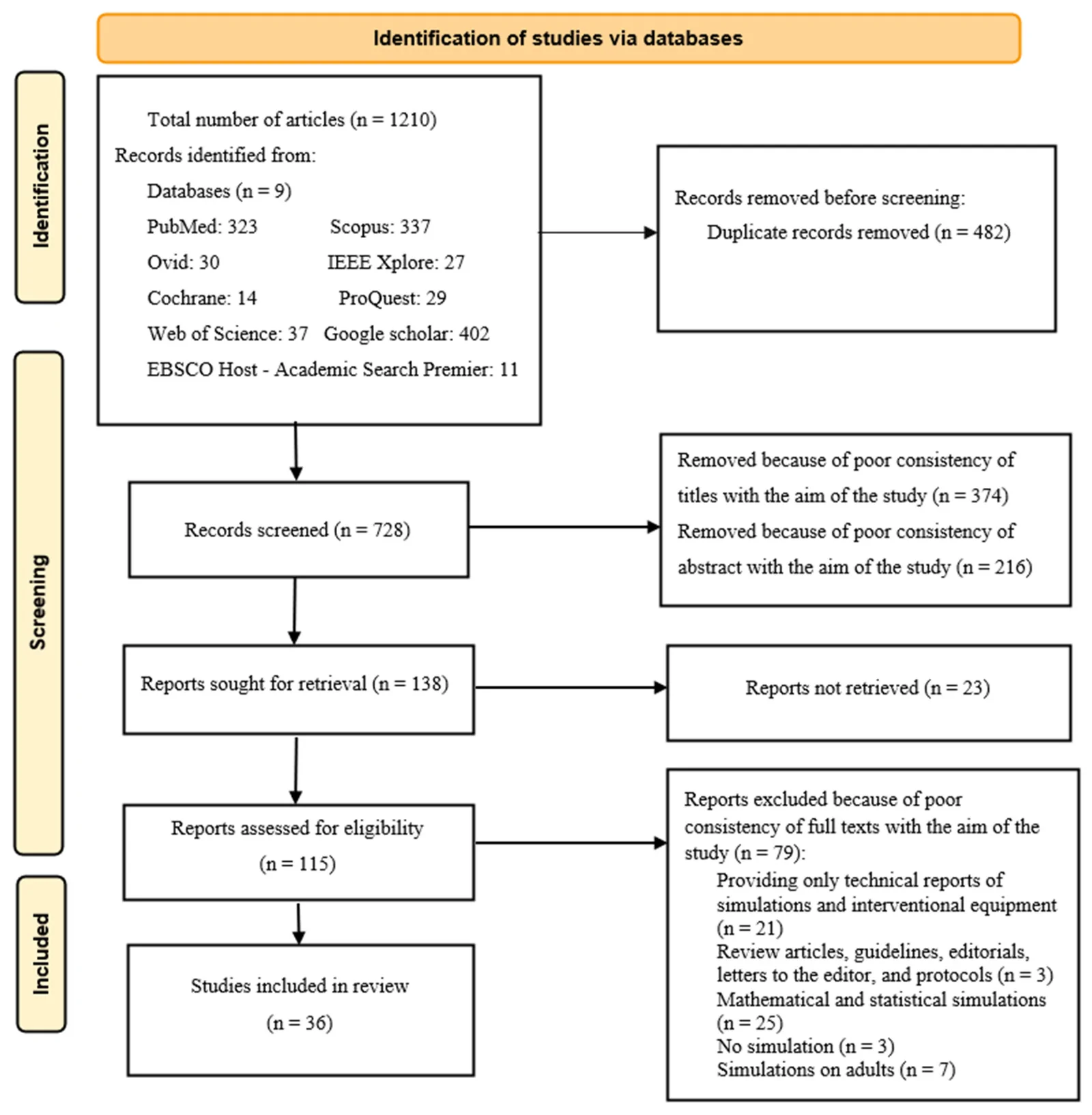
Process of article selection.

This systematic review included all eligible studies published up to 1 September 2025. Ethical approval was obtained from the Ethics Committee of Iran University of Medical Sciences (IR.IUMS.REC.1402.393) before the commencement of the study. The review protocol was registered in PROSPERO (CRD420251157450). The PRISMA 2020 checklist is provided as a Supplementary Material, and the corresponding PRISMA flow diagram, as illustrated in Figure 1, was developed to enhance transparency of the study selection process. To enhance the descriptive synthesis and facilitate interpretation of the included evidence, descriptive visualizations were generated to summarize graphical representations of publication trends, geographical distribution of studies, clinical application domains, cardiovascular conditions, and technology utilization patterns. These visualizations are presented in Section 3.

### 2.1. Eligibility Criteria

To ensure the selection of the most relevant studies, inclusion and exclusion criteria were set. According to the inclusion criteria, articles that were published in English up to 1 September 2025 and whose full texts were available were considered. The restriction to English-language publications was imposed because of time and resource constraints; this limitation is acknowledged as a potential source of language bias and needs to be addressed in future research. Additionally, all quantitative and qualitative research articles that aimed to investigate the application of various extended reality technologies, including virtual simulations, VR, AR, MR, and web-based simulations, in the context of pediatric cardiovascular diseases were included in the study. Review articles, guidelines, editorials, letters to the editor, protocols, and articles without full-text access were excluded. Studies were also excluded when the technology was not applied to the clinical setting or validated using clinical data. Similarly, studies that focused exclusively on the mathematical formulations or computational fluid dynamics were excluded if they were not used in the clinical setting or did not pass validation using patient data.

Furthermore, studies that solely reported technical aspects of extended reality technologies and articles related to mathematical and statistical formulas of simulations were not included. In fact, purely computational or mathematical studies focused on algorithmic development or computational fluid dynamics that lacked immediate clinical validation were excluded.

This review was designed according to the following PICO framework: Population (children aged 0–18 years with cardiovascular diseases), Intervention (computer-based simulation), Comparator (standard training or conventional planning), and outcomes (surgical metrics, clinical decision-making, and educational performance).

### 2.2. Information Sources

Databases used for searching articles included PubMed, Scopus, Ovid, IEEE Xplore, the Cochrane Library, ProQuest, Web of Science, Google Scholar, and EBSCO Host-Academic Search Premier. Articles published up to 1 September 2025 were searched in these databases. To manage the large volume of results from multiple databases and reduce noise, database-specific filters (such as language, publication date, and document type) were applied at the initial search stage within each platform. The complete search strings and database-specific filters are presented in Supplementary Table S1. Duplicate resources were removed using EndNote software (version X21, Clarivate) and further verified manually. Potential overlap among patient cohorts from the same institutions was assessed by reviewing institutional identifiers, author lists, study periods, sample characteristics, and reported patient populations across the included studies, and no evidence of overlapping patient cohorts was identified.

### 2.3. Search Strategy

The search criteria included specific terms from three distinct domains: computer-based simulation, cardiovascular diseases, and children. For computer-based simulation, terms included AR, patient-specific modeling, VR, computer-based simulation, patient simulation, MR, educational VR, patient-specific computational modeling, and interactive learning. Cardiovascular diseases included terms like heart disease, cardiac disorder, and heart defect. The synonyms used for children were child and pediatrics. Although terms related to congenital and structural cardiac conditions were included due to their frequent use in the simulation literature, broader cardiovascular terminology was utilized to maximize retrieval of studies across pediatric cardiovascular fields. This inclusive approach was adopted to reflect the full technological landscape within the defined scope of pediatric cardiovascular diseases. Supplementary Table S1 shows the detailed search strategies, including the applied filters and limits for each database.

### 2.4. Selection Process

The screening process followed the guidelines outlined in the Preferred Reporting Items for Systematic Reviews and Meta-Analyses (PRISMA 2020) statement [20]. After retrieving relevant articles, duplicates were removed using EndNote software (version X21, Clarivate). Two authors (A. Ab and A. Am) independently reviewed the titles and abstracts to assess relevance. Disagreements at this stage were limited and mainly related to whether studies aligned with the inclusion criteria, such as the use of 3D simulations or immersive technologies in pediatric cardiovascular diseases. A third author (H.A) was consulted to make the final decision when necessary. Full texts of potentially relevant studies were then independently reviewed by the same two authors to confirm eligibility. Disagreements during this phase usually concerned the scope of interventions or type of reported simulation and were resolved through discussion or consultation with the third author (H.A). The complete screening process is summarized in a PRISMA flow diagram (Figure 1).

### 2.5. Data Collection Process

Data were collected using a structured data extraction form by one of the authors (A. Ab), and the accuracy and completeness of the extracted data were verified by another author (H.A). Extracted data included author(s’) name(s), publication year, country, research objective, research methodology, disease type, application of computer-based simulation, type of technology, and a summary of the study results.

### 2.6. Data Items

Data were extracted using a data extraction form developed specifically for this systematic review. First, study-level characteristics were recorded, including the first author’s name, publication year, country of origin, study objective, and research design (such as quantitative cohort, cross-sectional survey, case series, or quasi-experimental design). Methodological features, comprising the sample size and the specific study setting (such as a tertiary cardiac center, university medical center, or medical school), were also captured. Second, the clinical and application contexts were documented. This included the underlying cardiovascular condition or specific diagnosis, CHDs such as tetralogy of Fallot or transposition of the great arteries, and the primary purpose of the computer-based simulation implementation. The application purposes were categorized based on the original studies and included domains such as preoperative surgical planning, intraoperative guidance, anatomical visualization, medical education and training, or clinical decision-making.

Third, the characteristics of simulation technology were extracted. This comprised the type of technology employed (VR, AR, MR, or 3D modeling software) and the specific hardware and software platforms utilized in each study (e.g., Microsoft HoloLens 2, Meta Quest Pro^®^ headset, Elucis™ software, Unity, 3D Slicer, or Stanford Virtual Heart software). Finally, the primary reported outcomes and key findings were extracted from each study. This included both quantitative measures, such as descriptive statistics, and qualitative or feasibility data, including user satisfaction, usability, implementation challenges, and technical limitations, as reported by the original authors. All extracted data were organized into summary tables to facilitate narrative synthesis and cross-study comparison.

### 2.7. Quality Assessment

The methodological quality of the included studies was appraised using the latest version of the Mixed Methods Appraisal Tool (MMAT) [21], which evaluates mixed-methods, qualitative, and quantitative studies across design-specific methodological criteria, including sampling strategy, data collection, and analytical rigor. Because the MMAT developers discourage calculation of an overall quality score, the original item-level assessments are presented. For descriptive comparison across studies, the proportion of fulfilled applicable criteria (Yes responses) was additionally calculated and classified into four descriptive categories: poor (≤0.40), moderate (0.41–0.59), good (0.60–0.79), and excellent (≥0.80). These categories were used solely to facilitate comparison across studies and do not represent official MMAT quality ratings.

For case reports, the Joanna Briggs Institute (JBI) Critical Appraisal Checklist for Case Reports was applied [22]. Each item was rated as Yes, No, Unclear, or Not Applicable. Similarly to the MMAT, overall numerical scores are not formally recommended by the JBI. Therefore, item-level assessments are presented, while the proportion of fulfilled criteria was additionally calculated to provide a descriptive summary and categorized as low (≤0.50), moderate (0.51–0.85), or high (≥0.85). These categories were used for descriptive purposes only.

### 2.8. Study Risk-of-Bias Assessment

Two authors (A. Ab and H.A.) independently assessed the risk of bias of the included studies. For quantitative studies, the Risk Of Bias In Non-randomized Studies of Interventions (ROBINS-I) tool was used [23] to evaluate bias across seven domains: confounding, selection of participants, classification of interventions, deviations from intended interventions, missing data, measurement of outcomes, and selection of the reported result. Based on these domains, each study was assigned an overall judgment of low, moderate, serious, or critical risk of bias. For qualitative studies, the Critical Appraisal Skills Programme (CASP) Checklist was used [24]. The CASP checklist comprises ten questions evaluating methodological rigor, credibility, and relevance of qualitative research, with each item rated as Yes, No, or Can’t tell. Disagreements between reviewers were resolved through discussion until a consensus was reached.

## 3. Results

### 3.1. Study Selection and Characteristics

A total of 1210 records were identified through searching nine databases (Figure 1). After removing duplicate records (n = 482), 728 records remained for screening. During title and abstract screening, 374 records were excluded because their titles were not relevant to computer-based simulation or pediatric cardiovascular diseases, and 216 records were excluded due to irrelevant abstracts, as they were not related to pediatric cardiovascular diseases or computer-based simulations, used simulations other than computer-based simulation, discussed mathematical or computational models (e.g., hemodynamic simulation) without a tangible 3D simulation, or involved only 2D image processing or enhancement without creating a 3D reconstructed, interactive, or immersive model, leaving 138 reports for full-text retrieval. Of these, the full texts of 23 articles could not be retrieved. The full texts of 115 articles were assessed for eligibility, resulting in the exclusion of 79 articles. Ultimately, 36 studies met the inclusion criteria and were included in the final review.

**Figure 2:**
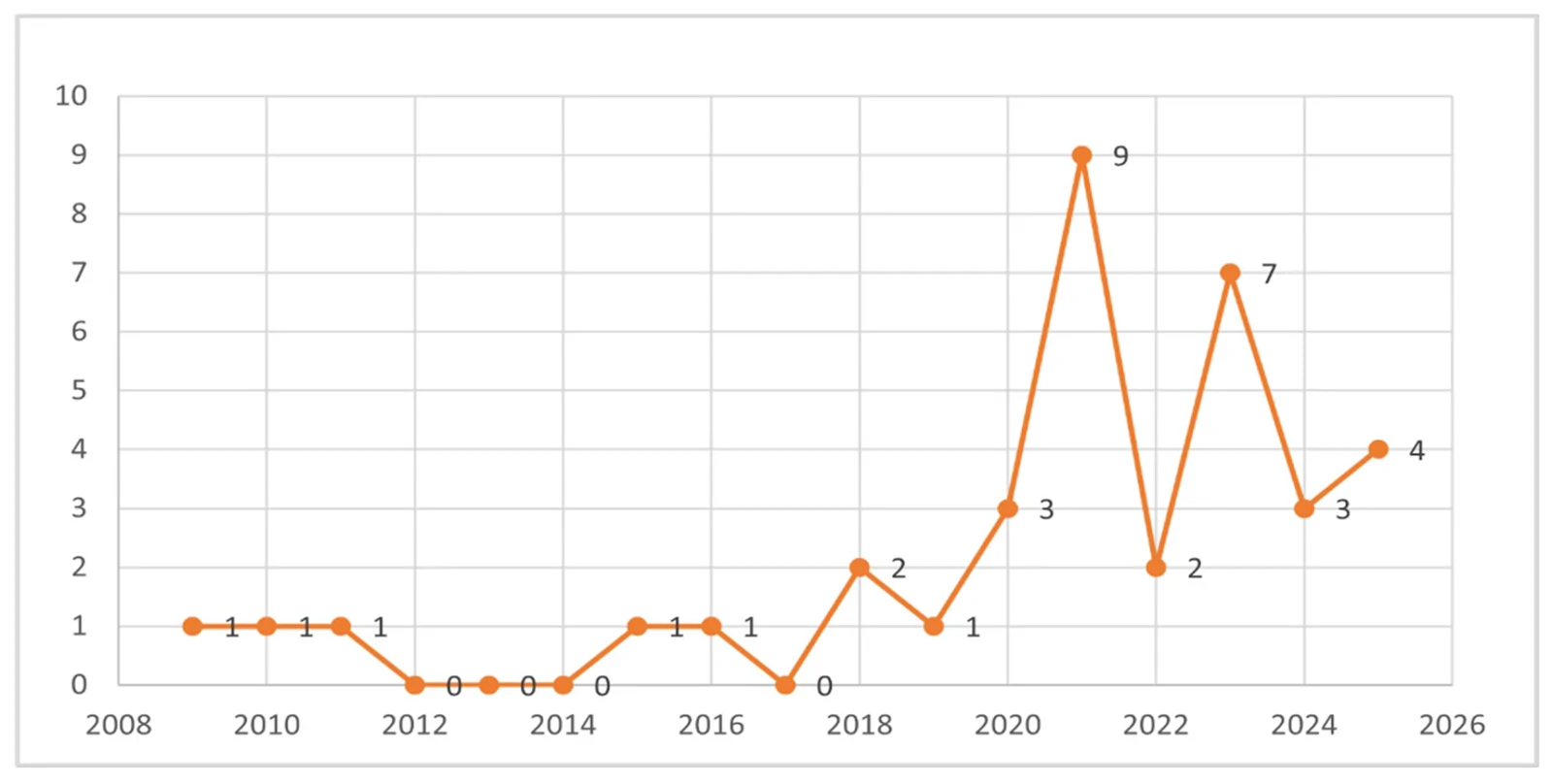
Frequency distribution of the studies by the year of publication.

The findings revealed that the publication period of the selected studies ranged from 2009 to 2025. The included studies were published in 2025 [25,26,27,28], 2024 [29,30,31], 2023 [32,33,34,35,36,37,38], 2022 [9,12], 2021 [8,39,40,41,42,43,44,45,46], 2020 [47,48,49], 2019 [50], 2018 [7,51], 2016 [52], 2015 [53], 2011 [54], 2010 [55], and 2009 [56]. The frequency distribution of these publications is shown in Figure 2.

**Figure 3:**
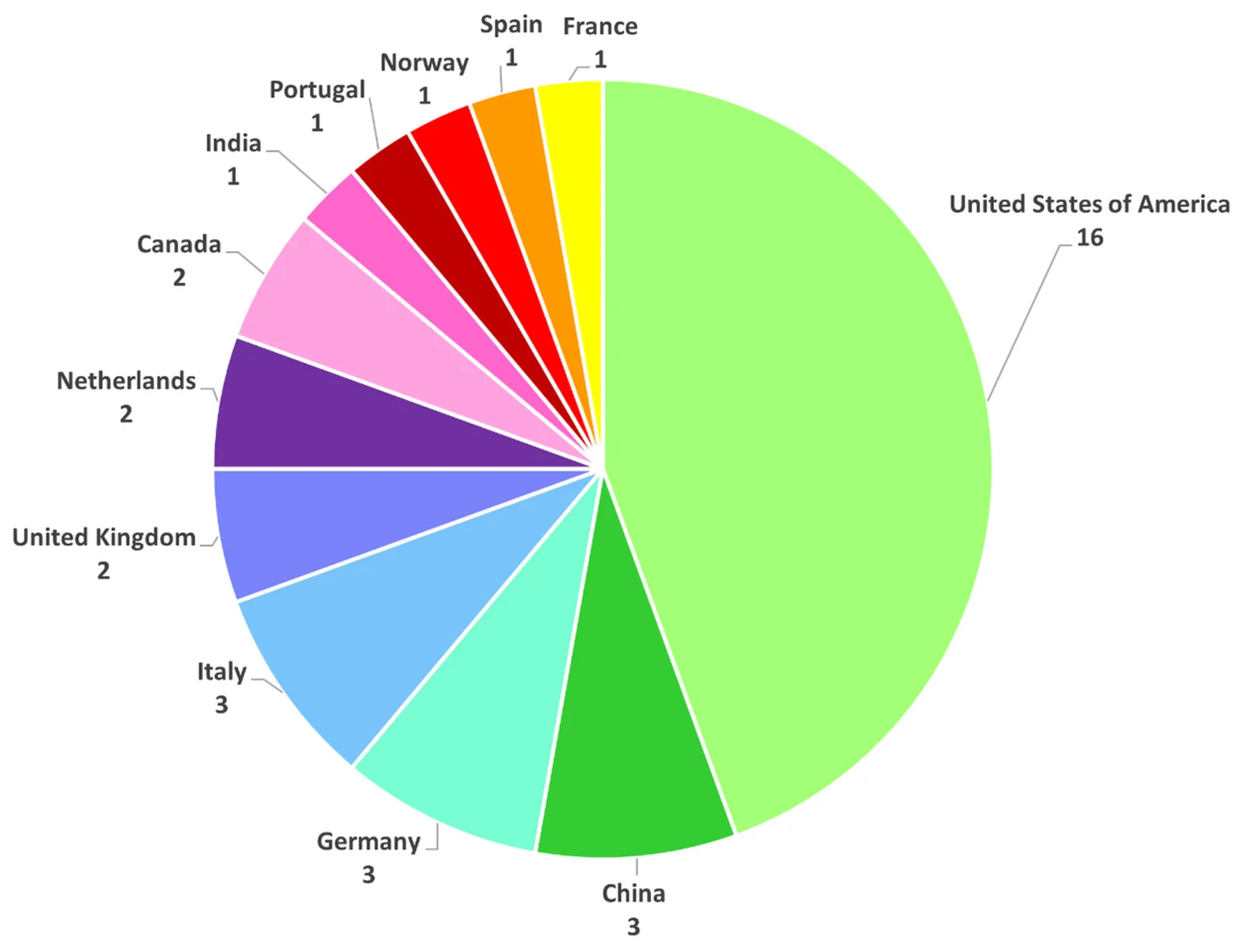
Geographical distribution of the selected studies.

The selected studies were conducted in various countries, including the United States (n = 16); China, Germany, and Italy (each with three studies); the United Kingdom, the Netherlands, and Canada (each with two studies); and India, Portugal, Norway, Spain, and France (each with one study). Figure 3 illustrates the geographical distribution of the selected studies.

Most of the included studies (33 out of 36) used quantitative approaches [7,8,9,12,25,26,27,28,29,32,33,34,35,36,37,38,39,40,41,42,43,44,46,47,48,49,50,51,52,53,54,55,56], while two studies adopted mixed-methods methodologies [30,31] and one study used a qualitative approach [45]. According to the results, various computer-based simulation technologies were used for surgical planning, diagnosis, and training in pediatric cardiovascular diseases. A summary of the research findings is presented in Supplementary Table S2.

### 3.2. Methodological Quality Assessment and Risk of Bias in the Studies

Based on the quality assessment of the included studies, a total of 15 quantitative non-randomized studies, 17 quantitative descriptive studies, two mixed-methods studies, one qualitative study, and one case report were evaluated. Among the quantitative non-randomized studies, scores ranged from 0.57 to 0.85, with four studies rated as moderate quality, five studies as good quality, and six studies achieving excellent quality. This trend reflects an increasing methodological rigor in more recent publications. For quantitative descriptive studies, scores ranged from 0.57 to 0.85, with six studies rated as moderate, 10 studies rated as good, and one study rated as excellent. Among mixed-methods studies, one study was rated excellent, while one was rated good, indicating generally high methodological quality for this design. The single case report achieved excellent quality, reflecting high methodological rigor across all assessed domains (Supplementary Table S3).

**Figure 4:**
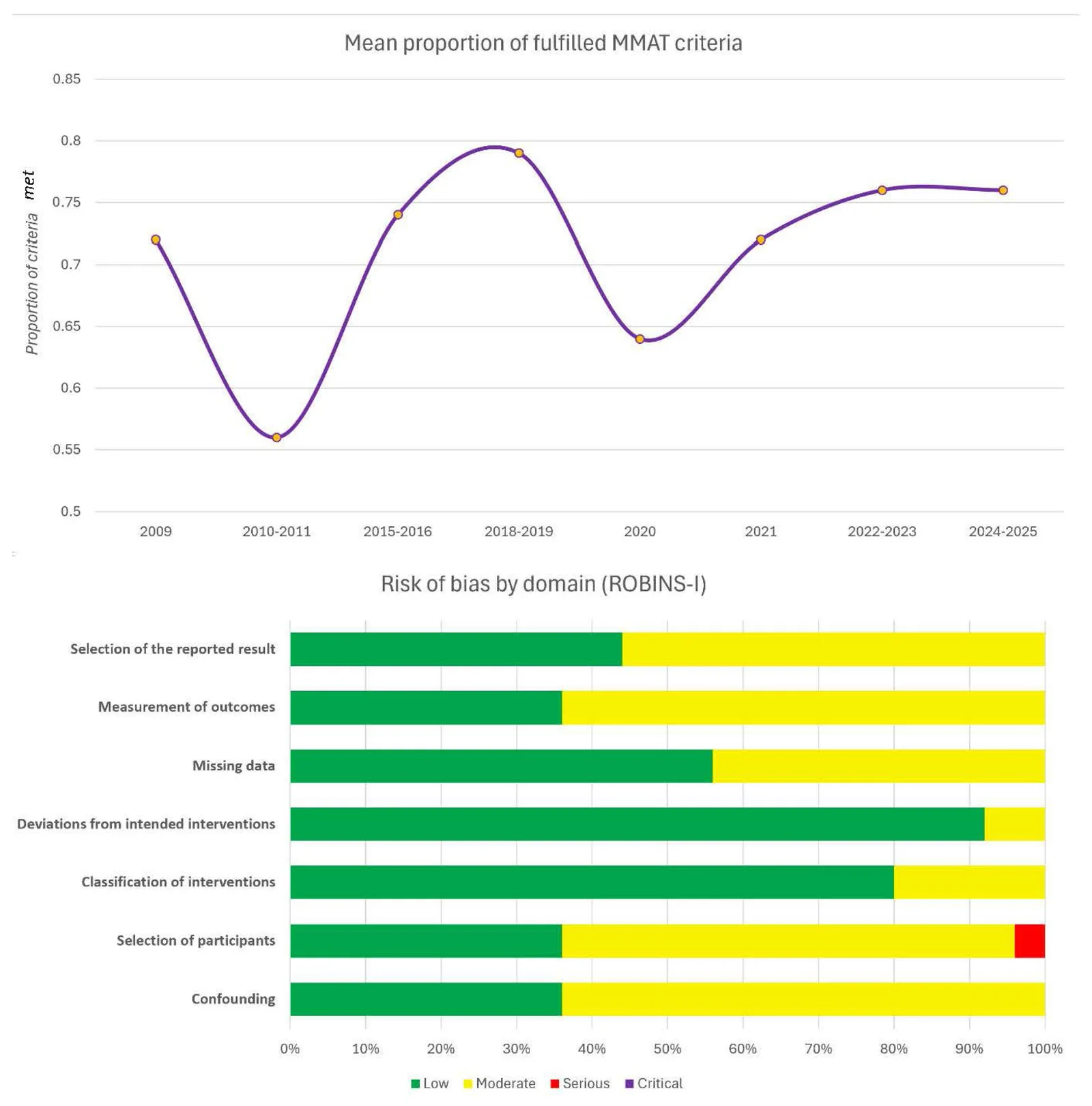
Methodological quality and risk of bias assessment of the fulfilled MMAT criteria.

Figure 4 presents the methodological quality and risk-of-bias assessment of the included studies. Panel A illustrates the mean proportion of the fulfilled MMAT criteria across publication periods, ranging from 0.56 (2010–2011) to 0.79 (2018–2019), with an overall mean of 0.71. Panel B displays the distribution of ROBINS-I risk-of-bias levels across seven domains, showing that the majority of studies had low-to-moderate risk, with serious risk observed primarily in the confounding and selection of participant domains.

The risk-of-bias assessment was conducted using the CASP checklist for a qualitative study and the ROBINS-I tool for quantitative studies. Qualitative studies showed a medium risk of bias, and quantitative studies primarily exhibited low-to-moderate overall risk of bias, though several studies showed serious risk in specific domains. Low risk of bias was commonly observed in outcome measurement and reporting, while moderate risk often appeared in confounding variables, participant selection, and deviations from the intended interventions. Serious risk was less frequent but notable in certain studies, highlighting vulnerabilities in the study design or data completeness. Most studies maintained acceptable methodological rigor, though a subset presented higher risk, underscoring the need for cautious interpretation of cumulative evidence (Supplementary Table S3).

### 3.3. Characteristics of the Included Studies

A summary of the key characteristics of all 36 included studies, including their design, sample size, applied technology, and primary findings, is presented in Table 1. A more detailed version of this table is available in Supplementary Tables S2 and S3.

### 3.4. Applications of Computer-Based Simulation Technologies

Computer-based simulation technologies were predominantly used for complex lesions that required detailed three-dimensional visualization. The most frequently investigated conditions were CHDs (n = 18) [7,8,9,12,25,26,28,29,30,35,36,38,44,45,46,51,55,56], double outlet right ventricle (DORV) (n = 6) [34,39,40,42,50,56], tetralogy of Fallot (TOF) (n = 5) [38,47,53,55,56], and ventricular septal defect (VSD) (n = 5) [28,43,48,52,55]. Other applications included MAPCAs, pulmonary atresia, AVSD, ccTGA, ASD, heart failure, valvular disorders, and several less common congenital and postoperative conditions [27,31,32,33,37,52,54].

Five categories of simulation technologies included VR [8,9,12,25,28,36,40,41,42,43,45,46,48,51], AR [26,29,30], MR [27,39,44,47,49,50], 3D modeling techniques [7,32,35,52,53,54,55,56], and multimodal technologies in which two or more approaches were combined [9,31,33,34,37,38,42,46,48]. VR was the predominant modality, mainly supporting surgical planning and education, whereas AR and MR were increasingly used for interactive visualization and collaborative decision-making.

**Figure 5:**
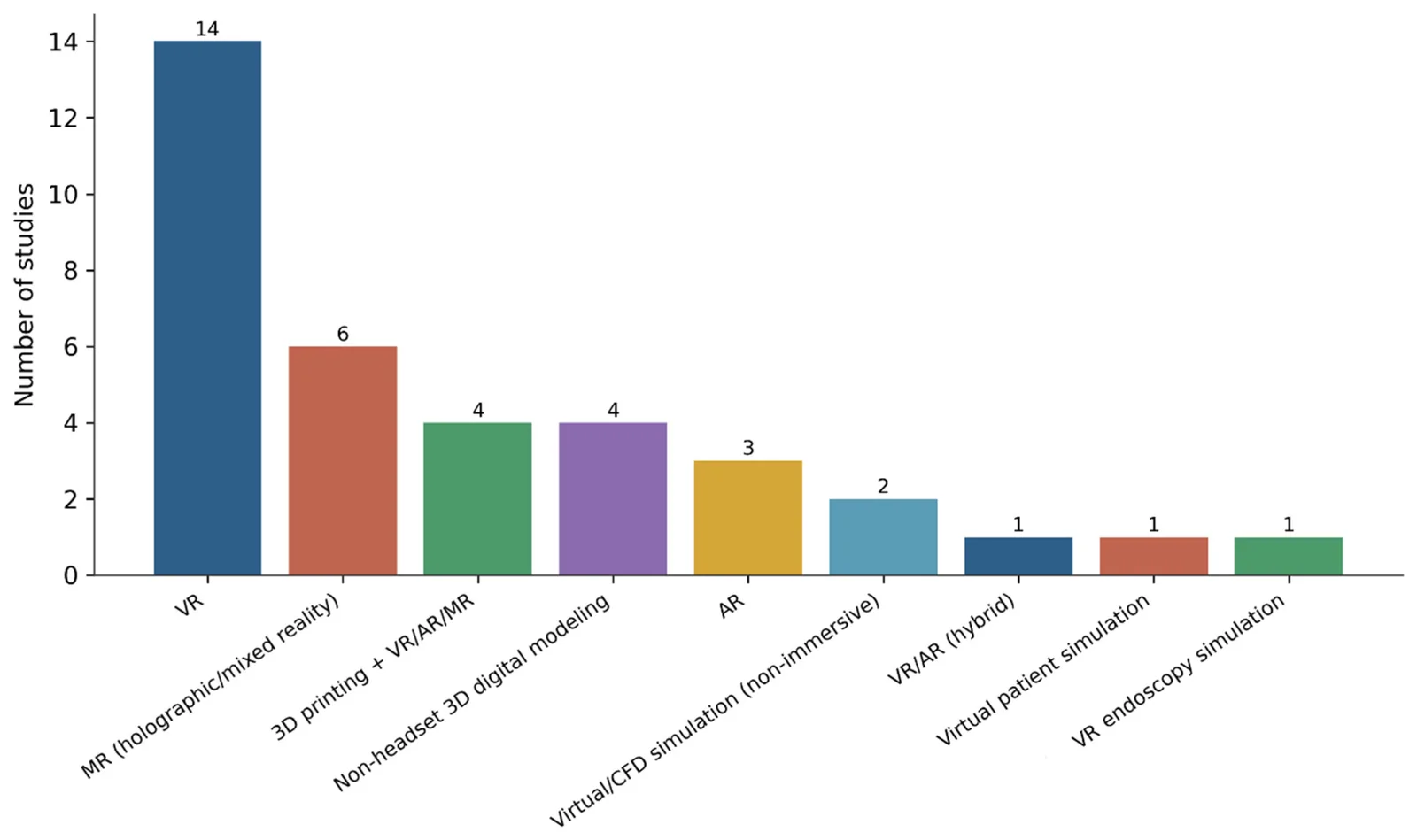
Distribution of computer-based simulation technologies.

**Figure 6:**
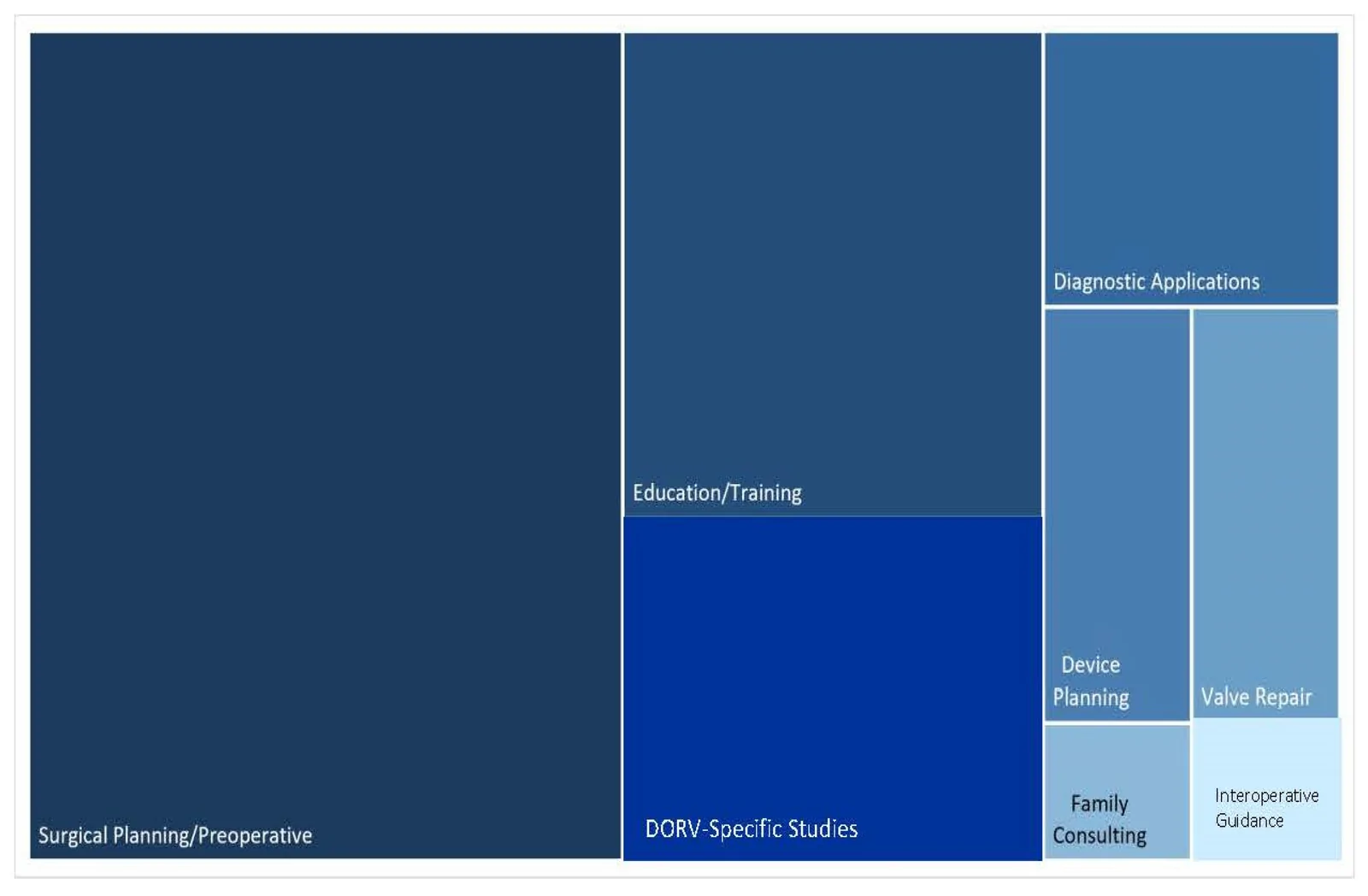
Distribution of clinical application domains across the included studies.

Figure 5 presents the distribution of various computer-based simulation technologies identified in the included studies, and Figure 6 shows the distribution of clinical application domains in the selected studies.

#### 3.4.1. Surgical Planning and Intraoperative Guidance

The findings showed that computer-based simulation was predominantly used for preoperative planning of complex congenital cardiovascular conditions. VR, MR, AR, and patient-specific 3D models enhanced visualization of spatial cardiac anatomy and provided additional information beyond conventional imaging modalities [8,9,12,25,27,30,31,32,33,34,35,39,40,41,42,44,45,46,47,48,50,51]. However, evidence regarding changes in surgical outcomes remains limited, as most studies were small observational cohorts [25,30,33,34,39,43,46,50,51,53].

A commonly reported benefit was enhanced visualization of spatial cardiac anatomy [12,48,51], often providing information beyond conventional imaging. For specific lesions such as DORV, VR and MR facilitated more detailed assessment of complex anatomy and supported selection of candidates for biventricular repair, with one study reporting 95% accuracy in identifying suitable candidates [39,42]. These technologies also supported virtual baffle planning and refinement of surgical strategies [40,42,50]. In pulmonary atresia with MAPCAs, computer-based simulation provided additional anatomical insights and supported preoperative planning [41,46]. In one study, MR-based visualization was also associated with reduced intraoperative preparation time [44]. Similarly, virtual simulation supported hemodynamic assessment in TOF with LPA stenosis [53]. While these findings were promising, they originated predominantly from small observational cohorts, limiting generalizability [39,40,41,42,44,46,50,53].

MR holography supported both preoperative and intraoperative guidance in complex CHD cases, including PAPVC, MAPCAs, right atrial isomerism with dextrocardia, and failing Fontan [27]. Interactive holographic models enabled surgeons to simulate surgical approaches, including vein redirection, Fontan pathway planning, coronary reimplantation, and unifocalization [27]. Despite these advantages, device bulkiness, manual calibration, and challenges in visualizing mobile structures may limit routine intraoperative adoption [27].

Mobile AR platforms enabled portable, collaborative, real-time planning [30], while multimodal simulations aided complex interventions, including MitraClip TEER in high-risk CHDs [31] and HeartMate3 implantation [32] in small children. Combined VR-, AR-, and 3D printing-optimized device implantation and could redirect strategies toward transplantation in adult heart failure [33]. In congenital cases, VR and 3D printing enhanced planning for DORV, especially VSD closure, with surgical plans based on 3D VR (80% concordance) and 3D printing (78%) matching the actual performed surgery more closely than plans based on 2D imaging alone (66%); 3D VR was particularly effective for assessing VSD patch-closure feasibility specifically (92% vs. 66% for 3D print and 46% for 2D) [34]. Virtual CT fitting also confirmed the feasibility of pediatric artificial heart pumps in small thoracic cavities [35].

#### 3.4.2. Medical Education and Training

For medical education, immersive technologies, particularly VR, were used to enhance understanding of cardiac anatomy and congenital heart defects [12,39,52]. The Stanford Virtual Heart curriculum was associated with significantly higher CHD knowledge scores among medical students and pediatric residents compared with conventional instruction, with high usability and engagement despite some reports of motion sickness [26,28,36]. VR was also preferred over 3D-printed models for anatomical understanding, with participants reporting higher understanding scores (8.5 vs. 6.3 out of 10) and 87% preferring VR, largely due to its dynamic and multiplanar visualization capabilities [38]. In addition, a web-based virtual patient simulation significantly improved medical students’ ability to distinguish normal from pathological heart sounds, with a 16% improvement following a short training workshop [52]. Overall, these findings suggested that computer-based simulation technologies may enhance cardiovascular education. However, differences in curricula, study designs, and assessment methods limit direct comparisons across studies [26,28,30,36,38].

#### 3.4.3. Diagnostic Support and Clinical Decision-Making

Computer-based simulation technologies contributed to diagnostic accuracy and clinical decision-making in pediatric cardiovascular diseases. MR holographic guidance (MRHG) achieved 100% accuracy in DORV-type diagnosis, compared to 88.2% with conventional methods, and improved accompanying-malformation diagnostic accuracy (95.5% vs. 89.7%), while also reducing surgical planning time (51.65 ± 11.11 min vs. 65.71 ± 18.07 min) [39]; similarly, a study demonstrated high diagnostic accuracy (98.7% for ASD) using virtual endoscopy [55].

Patient-specific VR models also influenced clinical decision-making. In a study of 10 patients with complex CHDs, VR-based visualization changed management decisions in seven patients by providing additional anatomical information beyond conventional CT imaging [25]. Similarly, MR provided an immersive and interactive three-dimensional representation of complex cardiac anatomy and supported simulation of surgical approaches in conditions such as PAPVC and failing Fontan, with reported benefits for intraoperative guidance and accuracy [27].

The feasibility of integrating simulation technologies into clinical decision-making was further demonstrated by Bertelli et al., who showed that accurate patient-specific 3D reconstructions could be generated rapidly, including by inexperienced users, supporting their potential use in clinical settings [37]. Similarly, a multimodal protocol combining virtual and physical simulation supported preprocedural planning in four high-risk patients with CHDs and complex anatomies undergoing transcatheter edge-to-edge repair [31]. These findings suggest that computer-based simulation technologies can enhance anatomical understanding, support diagnostic assessment, and provide additional information for clinical decision-making.

#### 3.4.4. Resource Optimization and Implementation Challenges

Some studies compared the outcomes of using computer-based simulation in relation to resource utilization and implementation compared to traditional methods [7,44]. MR holograms were considered cost-effective for diagnosing complex CHDs. Unlike 3D prints, holographic models allow for rapid modifications using original images and echocardiographic data, providing dynamic visualization [44,50]. Thirty-six pediatric cardiology team members evaluated MR holograms using a diagnostic questionnaire and quality ratings and considered positive scores (5.32 to 5.46) for a favorable 3D experience, good understanding of morphology, effective sharing features, and MR functionality [50].

VR 4D heart models were more cost-effective than 3D-printed models, as they used open-source software, required no physical materials, and allowed easy manipulation, benefiting medical education and surgical planning [47]. Awori et al. found that VR was significantly more effective for education than 3D prints, with participants demonstrating a better understanding of cardiac anatomy (8.5/10 vs. 6.3/10) and 87% preferring VR. A key cost-saving advantage is that VR provides dynamic, multiplanar visualization from a single digital model, eliminating the material cost and need to produce multiple physical models for different views [38].

Despite these potential advantages, implementation challenges remained evident. Hardware limitations, device bulkiness, calibration requirements, motion sickness, and the need for user training may limit routine adoption [26,27,28,30,39]. Successful integration into clinical and educational workflows also requires appropriate technical infrastructure, portability, and user familiarity [26,30]. These findings indicated that implementation should consider not only technical performance but also usability, workflow integration, training requirements, and resource implications.

### 3.5. Challenges and Limitations Reported in Included Studies

Several studies identified technical challenges associated with the implementation of computer-based simulation technologies, including image processing time, calibration difficulties, hardware limitations (e.g., device bulkiness and manual calibration), motion sickness among VR users, and software dependency [28,36,37,41,44,47,49,50]. Van de Woestijne et al. reported prolonged evaluation times, setup complexity, and limited image loading during the use of 3D-VR imaging for preoperative planning [41]. Similarly, Blonsky et al. observed occasional motion sickness among VR users, although this did not substantially affect overall user satisfaction [28].

Barriers to clinical implementation were also frequently reported. Challenges related to workflow integration, the need for trained personnel, and the limited availability of simulation technologies were identified as factors restricting routine clinical adoption [26,28,30,41]. Brun et al. noted that implementation of MR holograms required considerable investment in infrastructure and user training despite favorable user acceptance [50]. Likewise, technical constraints, including device bulkiness, manual calibration, and difficulties in tracking mobile anatomical structures, limited the routine intraoperative use of MR systems [27].

Methodological limitations were common across the included studies. Most investigations were small-sample, single-center observational studies with limited long-term outcome evaluation [8,12,26,27,28,29,32,33,34,35,36,37,38,39,40,41,42,43,44,46,47,48,49,50,51,52,53,54,55,56]. The qualitative study demonstrated a moderate risk of bias, whereas quantitative studies generally showed low-to-moderate overall risk of bias, with serious concerns in specific domains, particularly confounding, participant selection, and deviations from intended interventions (Supplementary Table S3). Consequently, the predominantly observational study designs limit the ability to establish causal relationships between the use of simulation technologies and clinical outcomes.

In addition to these methodological limitations, several studies reported neutral or inconclusive findings [34,39]. Although improvements in anatomical visualization and user experience were consistently described, long-term clinical outcome data were generally lacking, and a small number of studies reported limited advantages over conventional imaging in selected clinical scenarios [29,34]. Furthermore, the available evidence was derived predominantly from small observational cohorts, limiting the generalizability of findings and precluding definitive conclusions regarding the superiority of any individual simulation modality [7,8,12,25,27,30,31,32,33,34,37,40,41,42,43,46,47,48,49,50,51,53,54,55,56].

### 3.6. Synthesis

This systematic review demonstrated that computer-based simulation technologies were primarily investigated for preoperative planning, medical education, diagnostic support, and clinical decision-making in pediatric cardiovascular diseases. Overall, VR, AR, MR, and three-dimensional modeling consistently improved visualization of complex cardiac anatomy and facilitated communication among clinicians, trainees, and multidisciplinary teams. These technologies were most frequently applied to congenital heart diseases, particularly complex CHDs, DORV, and TOF. Educational studies generally reported improved anatomical understanding, learner engagement, and knowledge acquisition compared with conventional teaching approaches, whereas clinical studies suggested potential benefits for surgical planning, diagnostic confidence, and procedural decision-making. The strength of evidence remains limited because most studies were observational, conducted at single centers, included relatively small sample sizes, and rarely evaluated long-term clinical outcomes. The available evidence supported the feasibility and potential clinical utility of computer-based simulation technologies in pediatric cardiovascular care. Nevertheless, larger multicenter studies using standard methodologies and clinically relevant outcome measures are needed to determine their effectiveness and establish their role in routine clinical practice.

It is notable that a quantitative meta-analysis was not undertaken. In fact, as no pooled statistical analysis was performed, formal heterogeneity statistics such as I^2^ or Cochrane’s Q were not calculated. The included studies varied considerably in patient populations, underlying cardiovascular conditions, simulation platforms (VR, AR, MR, and 3D modeling), comparator methods, study designs, and outcome measures, which made meaningful statistical pooling inappropriate. A narrative synthesis was therefore undertaken to summarize and integrate the available evidence.

## 4. Discussion

### 4.1. Principal Findings

This systematic review synthesized evidence from 36 studies in which computer-based simulation technologies were assessed in pediatric cardiovascular diseases. These technologies were applied to anatomical visualization, procedural planning, diagnostic support, and medical education, particularly for complex CHDs, and were reported as complementary rather than standalone tools. High diagnostic performance was reported for selected approaches, including virtual endoscopy [55] and MR holography [39], and positive educational outcomes were reported for several studies [12,28,36,38,39,52]. However, these findings should be interpreted cautiously, as they derived largely from small, single-center, observational cohorts, with limited long-term clinical outcome data. So, it seems that they support feasibility and short-term benefits rather than established clinical superiority.

### 4.2. Comparison with Previous Literature

Previous systematic reviews have consistently reported that computer-based simulation and XR technologies show promise for improving anatomical visualization, procedural planning, and medical education in cardiovascular care, although the available evidence remains heterogeneous and is largely derived from observational studies and feasibility reports. For example, Bouraghi et al. [57] categorized VR applications into physical rehabilitation, psychological rehabilitation, and education/training and concluded that VR may improve technical performance, procedural efficiency, and learning outcomes while emphasizing the need for larger studies with longer follow-up. Similarly, Kanschik et al. [58] identified peri-procedural planning and education as the predominant applications of XR in a large systematic review of XR in cardiovascular medicine (164 studies) but concluded that robust randomized controlled trials remain necessary before these technologies can be routinely integrated into clinical practice. These conclusions are consistent with the findings of the present review, in which educational benefits and potential improvements in procedural planning were frequently reported. However, high-quality evidence supporting meaningful improvements in clinical outcomes remains limited.

Previous narrative reviews focusing on congenital heart disease have likewise highlighted the value of patient-specific three-dimensional visualization for surgical planning, trainee education, and procedural guidance. Salavitabar et al. [11] described emerging 3D technologies as promising tools for teaching, prediction, procedural planning, and intraoperative guidance in congenital heart disease, whereas Gunsaulus et al. [59] emphasized the growing role of XR in improving family education, trainee understanding, and preprocedural decision-making for complex congenital heart disease. Our findings support these observations by demonstrating that the use of computer-based simulation technologies in pediatric cardiovascular diseases may provide potential benefits across multiple user groups and clinical contexts.

It is notable that, unlike previous reviews that primarily summarized the potential applications of individual technologies or focused on specific clinical domains [11,57,58,59], the present review provided a broader critical synthesis of the available evidence in pediatric cardiovascular diseases. In addition to summarizing clinical and educational applications, we systematically evaluated methodological quality, evidence heterogeneity, implementation challenges, patient- and family-centered considerations, and research gaps. Our findings further demonstrated that the current evidence remains disproportionately concentrated on VR and 3D modeling, whereas AR and MR continue to be supported mainly by small feasibility studies. This observation is consistent with the interpretation of Kanschik et al. [58], who noted the relative scarcity of robust evidence for AR and MR applications. Furthermore, recent commentary by Sun and Vaccarezza [60] reinforced that although XR technologies have considerable potential across cardiovascular medicine, their widespread clinical adoption will depend on stronger evidence from well-designed comparative studies. Overall, these findings indicate that the field is progressing rapidly, but the transition from technological feasibility to evidence-based clinical implementation has not yet been fully achieved.

### 4.3. Clinical Applications and Interpretation of Findings

The clinical applications consisted of three related domains: diagnostic support, procedural planning, and medical education. For diagnostic support, MR holography and virtual endoscopy showed high accuracy for selected congenital lesions [39,55], and patient-specific VR models influenced management decisions in complex CHDs [25]. The results supported the main value of simulation in improving interpretation of existing imaging data, but it is not clear whether these technologies consistently improve diagnostic accuracy and treatment selection in other diseases or not.

For procedural planning, VR, MR, AR, and patient-specific 3D models supported spatial orientation, complex approaches, and multidisciplinary communication, notably in DORV [9,39,40,42,50] and pulmonary atresia with MAPCAs [41,46]. MR holography also supported intraoperative guidance in cases such as PAPVC and failing Fontan [27]. These findings reflect improved visualization and planning capability rather than a proven surgical outcome benefit, since most studies lacked control groups or long-term follow-up on complications, reintervention, or survival.

For medical education, VR-based curricula, including Stanford Virtual Heart, improved knowledge scores among students and residents despite occasional motion sickness [26,28,36], and VR was preferred over 3D-printed models for anatomical understanding (8.5 vs. 6.3/10; preferred by 87%) [38]; a web-based simulator also improved auscultation accuracy by 16% [52]. These educational gains are consistent with cognitive load theory and deliberate-practice frameworks, which suggest that immersive 3D visualization reduces the effort needed to mentally reconstruct anatomy from 2D images and support scaffolded, feedback-driven skill development [17,18,61,62,63,64]. However, poorly designed interfaces or cybersickness can offset this benefit [61,65], and short-term knowledge gains do not guarantee retention or clinical performance [62]. Taken together, these findings support integrating simulation within structured curricula and planning workflows as a complement to, not a replacement for, conventional teaching and imaging methods [61,66].

### 4.4. Evidence Strength and Research Gaps

Despite promising applications, the current evidence remains preliminary. Most included studies were observational, feasibility studies, or educational evaluations with small sample sizes and heterogeneous outcomes. Risk-of-bias assessment showed various quality scores across studies, with concerns mainly related to confounding variables, participant selection, and deviations from intended interventions. The qualitative evidence was also limited by methodological concerns, with a moderate risk of bias.

Evidence was not evenly distributed across technologies. VR and 3D modeling accounted for most applications, whereas AR and MR were less frequently evaluated. This imbalance may reflect the relative maturity of each technology, with VR having more established platforms and a longer history of use in medical applications. It also limits the ability to make conclusions regarding the comparative effectiveness of different simulation modalities. Similarly, complex CHDs, such as TOF and DORV, were more commonly studied than other pediatric cardiovascular conditions, including acquired heart diseases. This indicated potential gaps in evidence generation.

A quantitative meta-analysis was precluded by substantial clinical and methodological heterogeneity across the included studies. Variation existed in patient populations, underlying cardiovascular conditions, simulation platforms, comparator methods, study designs, and outcome measures, making meaningful statistical pooling inappropriate. The absence of standardized outcome measures further limited the ability to compare effectiveness across studies. Future research should therefore prioritize validated and clinically meaningful outcomes, including diagnostic accuracy, procedural time, complications, reintervention, educational retention, and patient- and family-reported outcomes.

### 4.5. Patient and Family Perspectives

Beyond professional training, patient-specific simulation models may have implications for patient and family communication. Visualization of congenital cardiac abnormalities through interactive 3D models may improve parental understanding of disease anatomy and treatment options, potentially supporting shared decision-making and informed consent [54]. One included study reported that children and their families could visualize heart conditions in interactive models, facilitating the treatment process and reducing presurgical anxiety [54].

However, the evidence supporting these potential benefits remains extremely limited. No included studies systematically evaluated parental comprehension, decisional conflict, satisfaction with communication, or other validated patient- and family-reported outcomes. Thus, although patient-specific visualization may facilitate communication of complex anatomical and therapeutic information, its actual effect on shared decision-making and family experiences remains uncertain.

This represents an important gap because pediatric cardiovascular decisions frequently involve parents or caregivers, who must understand complex anatomical and therapeutic information while making decisions on behalf of children. Future studies should therefore evaluate simulation-based communication using validated measures of comprehension, confidence, decision conflicts, satisfaction, and anxiety rather than relying primarily on informal user perceptions. Ethical considerations, including issues related to informed consent, parental expectations, data privacy, and equitable access, have also received little attention in the existing literature. The use of highly realistic patient-specific models should support, rather than replace, clinician–family communication and should be accompanied by appropriate explanation of uncertainty and treatment alternatives.

### 4.6. Barriers and Implementation Challenges

Several barriers may limit routine clinical adoption of computer-based simulation technologies. The acquisition and maintenance costs of VR/MR hardware, specialized software, and personnel training may restrict implementation, particularly in resource-limited settings and smaller centers [26,28,41]. Integration into clinical workflows requires technical expertise, standard protocols, and interoperability with existing imaging systems [27,50].

Technical limitations were frequently reported across studies, including device bulkiness, manual calibration, image processing time, and motion sickness among VR users [28,36,37,41,44,47,49,50]. These challenges may affect user experience and restrict routine intraoperative use. Patient-specific models derived from imaging data raise concerns about data protection, storage, and sharing, particularly when using cloud-based platforms or multi-user AR environments [30]. Clinicians require dedicated training to use simulation technologies effectively, and the learning curve may be steep for those unfamiliar with immersive interfaces [26,50].

Implementation should also be considered in terms of sustainability rather than initial technical feasibility alone. Although some studies reported potential resource or cost advantages associated with digital models [7,47], robust comparative economic evaluations remain scarce. The costs of hardware acquisition, software development, maintenance, model generation, personnel training, and workflow adaptation should therefore be considered along with potential savings in physical model production, procedural preparation, or educational resources. The potential for over-reliance on simulation outputs and its effect on clinical reasoning requires further investigation. Unequal access to advanced simulation resources may exacerbate existing disparities in pediatric cardiovascular care between high-resource and low-resource settings. These barriers are not merely technical but also organizational and financial. Reimbursement mechanisms and long-term economic sustainability for simulation-guided care have not been adequately established, and the cost-effectiveness of these technologies compared with conventional approaches remains insufficiently evaluated. Addressing these barriers will require multidisciplinary collaboration among clinicians, engineers, educators, and health policymakers.

Overall, the available evidence suggests that although computer-based simulation technologies offer considerable promise for pediatric cardiovascular care, their successful translation into routine practice will depend not only on stronger clinical evidence but also on overcoming important methodological, organizational, financial, ethical, and equity-related challenges.

## 5. Research Limitations

This review had several methodological limitations. First, restriction to English-language publications may have resulted in language bias, as relevant studies published in other languages could have been excluded. Second, although nine databases were searched, some relevant studies may not have been identified, particularly those published in non-indexed journals or gray literature. Third, publication bias is possible, as studies reporting positive or favorable findings might be more likely to be published than studies reporting negative or inconclusive results. Formal assessment of publication bias was not feasible because quantitative pooling was not performed. Fourth, the review included studies with varying methodologies and quality, and despite using standard appraisal tools, the subjective nature of quality assessment may introduce some degree of interpretative bias.

Fifth, most included studies were observational, single-center investigations with small sample sizes, limiting the generalizability of findings and precluding causal inferences. Sixth, substantial clinical and methodological heterogeneity across studies prevented quantitative meta-analysis and limited the strength of pooled conclusions. Seventh, most studies evaluated feasibility, usability, or educational outcomes rather than clinically meaningful endpoints such as postoperative complications, reintervention rates, or patient-reported outcomes. Eighth, papers related to premature ventricular contraction (PVC) were not included in the current study, as PVC is not considered a structural heart disease and might be a disease symptom. Finally, risk-of-bias assessment identified serious concerns in several studies regarding confounding, participant selection, and deviations from intended interventions, further limiting confidence in causal claims.

## 6. Future Research Directions

Based on the findings and limitations of this review, several priorities for future research emerge. First, large multicenter randomized controlled trials or well-designed prospective comparative studies are needed to compare simulation-based planning with conventional imaging approaches using standard clinical outcomes, including surgical complications, reintervention rates, and long-term survival. The development and validation of core outcome sets for simulation research in pediatric cardiology would facilitate cross-study comparisons and future meta-analyses. In addition, comprehensive cost-effectiveness analyses are required to determine whether the clinical and educational benefits of simulation technologies justify their implementation, particularly in resource-constrained settings. Future studies should also incorporate patient- and parent-reported outcomes, including satisfaction, decisional conflict, anxiety, and quality of life, to better evaluate the patient-centered outcomes of simulation. Research focusing on implementation science is needed to identify effective strategies for integrating these technologies into routine clinical workflows while addressing training requirements and barriers to adoption. Furthermore, future investigations should explicitly consider ethical issues, including informed consent for minors, data privacy and security, the potential for over-reliance on technology, and equitable access across different socioeconomic and geographic settings. Finally, additional research is warranted in underrepresented areas, particularly acquired heart diseases and low-resource settings, where computer-based simulation technologies may provide potential clinical value.

## 7. Conclusions

This systematic review synthesized evidence from 36 studies in which computer-based simulation technologies were used and evaluated in pediatric cardiovascular diseases. The findings suggested that these technologies helped with diagnostic support, procedural planning, and medical education, particularly in complex congenital heart diseases. However, the available evidence remains preliminary and is characterized by small sample sizes, heterogeneous methodologies, and limited long-term evaluation of clinical outcomes. Therefore, computer-based simulation technologies should be considered complementary tools rather than replacements for conventional imaging, clinical expertise, or established educational approaches. Future multicenter comparative studies using standardized clinical, educational, patient-centered, and economic outcomes are needed to determine their added value and support evidence-based implementation in routine pediatric cardiovascular care.

## Figures and Tables

**Table 1:** Characteristics of the included studies.

Author/Year	Country	Disease/ Application	Technology	Study Design/ Sample Size	Main Outcome	Quality Assessment
Riesenkampff et al., 2009 [56]	Germany	Complex CHD	Virtual 3D models	Quantitative (n = 11)	Enhanced surgical decision-making and consensus	Good
Xue et al., 2010 [55]	China	ASD, VSD, TOF, DORV	Virtual endoscopy (3DE IESS)	Quantitative (n = 40)	High diagnostic accuracy (98.7% for ASD)	Moderate
De Zélicourt et al., 2011 [54]	USA	Single ventricle, PAVM	Virtual surgery simulation	Quantitative (n = 5)	Identified optimal baffle strategies	Moderate
Rao et al., 2015 [53]	USA	TOF, LPA stenosis	Virtual simulation	Quantitative (n = 2)	Improved hemodynamics in stenosis cases	Good
Pereira et al., 2016 [52]	Portugal	Cardiac pathologies	Virtual patient simulation	Quantitative (n = 117)	Improved auscultation skills (+16%)	Good
Olivieri et al., 2018 [7]	USA	CHD (ICU care)	Virtual 3D models (tablet)	Quantitative (n = 20)	Improved provider confidence and care delivery	Excellent
Ong et al., 2018 [51]	USA	Truncus Arteriosus, VSD	VR (HTC Vive)	Quantitative (n = 2)	Feasible and effective for presurgical planning	Good
Brun et al., 2019 [50]	Norway	DORV-TGA	MR (HoloLens)	Quantitative (n = 1)	Accurate diagnosis; positive user experience	Good
Capellini et al., 2020 [48]	Italy	CHD	VR + 3D printing	Quantitative (20 datasets)	Enhanced decision-making and confidence	Moderate
Priya et al., 2020 [47]	USA	TOF	VR 4D models	Quantitative (n = 1)	Dynamic interaction; cost-effective alternative	Moderate
Kang et al., 2020 [49]	Canada	DORV	MR (True 3D)	Quantitative (12 specimens)	Accurate stereoscopic morphological representation	Moderate
Huang et al., 2021 [45]	USA	CHD	Hybrid VR-desktop (PVH)	Qualitative (no patients)	Positive feedback for planning and consultation	Excellent
Cen et al., 2021 [46]	China	Pulmonary atresia, VSD, MAPCA	VR + MR + 3D printing	Quantitative (n = 5)	Improved surgical understanding and outcomes	Good
Gehrsitz et al., 2021 [44]	Germany	CHD	MR (CR-holograms)	Quantitative (n = 26)	Superior to 2D/3D printing; reduced prep time	Good
Milano et al., 2021 [42]	UK	DORV	VR (Oculus Rift)	Quantitative (n = 10)	Improved identification of biventricular repair candidates	Excellent
Pushparajah et al., 2021 [43]	UK	AVV disease	VR (HTC Vive)	Quantitative (n = 15)	Modified surgical approach in majority of cases	Good
Raimondi et al., 2021 [8]	France	CHD	VR (DIVA)	Quantitative (n = 3)	Improved visualization; faster processing	Moderate
van de Woestijne et al., 2021 [41]	Netherlands	Pulmonary atresia, MAPCA	3D-VR (CardioVR)	Quantitative (n = 7)	Provided additional anatomical insights	Moderate
Vigil et al., 2021 [40]	USA	DORV	VR (SlicerVR)	Quantitative (n = 3)	Streamlined virtual baffle planning	Excellent
Ye et al., 2021 [39]	China	DORV	MR (MRHG)	Quantitative (n = 34)	100% accuracy in DORV type diagnosis	Good
Ghosh et al., 2022 [9]	USA	CHD	VR + 3D modeling	Quantitative (112 cases)	Successfully integrated into clinical care workflow	Good
Vegulla et al., 2022 [12]	USA	CHD specimens	VR (HTC Vive)	Quantitative (7 specimens)	Enabled interactive exploration for education	Good
Stepanenko et al., 2023 [33]	Spain	Complex HF, Ebstein anomaly	VR/AR + 3D printing	Quantitative (n = 4)	Changed treatment approach; enhanced planning	Excellent
Italiano et al., 2023 [32]	Italy	Dilated cardiomyopathy	3D simulation	Case report (n = 1)	Enabled successful HM3 implant in a small child	Moderate
Peek et al., 2023 [34]	Netherlands	DORV	3D-VR + 3D printing	Quantitative (n = 5 patients, 12 participants)	VR plans matched actual surgery (80% vs. 66% for 2D)	Moderate
Miyagi et al., 2023 [35]	USA	CHD, DCM	Virtual 3D CT fitting	Quantitative (n = 40)	Assessed anatomical fit for pediatric TAH pumps	Good
Bertelli et al., 2023 [37]	Italy	Partial anomalous pulmonary venous connection (PAPVC)	VR (DIVA)	Quantitative (n = 5 patients, 5 participants)	Inexperienced users produced accurate models	Good
Awori et al., 2023 [38]	USA	TOF, normal heart	VR vs. 3D printing	Quantitative (n = 30)	VR perceived more effective than 3D-printed models	Good
Lim et al., 2023 [36]	USA	Common CHDs	VR curriculum	Quantitative (n = 142)	Significantly increased CHD knowledge scores	Excellent
Salavitabar et al., 2024 [29]	USA	Multiple CHDs	AR (HoloLens)	Quantitative (n = 30)	AR superior for 3D spatial understanding	Excellent
Mehta et al., 2024 [30]	USA	CHD	AR (ARCoLLAB)	Mixed methods (n = 5)	Highly rated for collaborative surgical planning	Good
Jolley et al., 2024 [31]	USA	AVVR in CHD	Virtual simulation	Case series (n = 4)	Successful TEER; significant AVVR reduction	Excellent
Blonsky et al., 2025 [28]	USA	Common CHDs	VR	Quantitative (n = 373 students)	Higher knowledge scores; >99% recommended integration	Excellent
Ponzoni et al., 2025 [27]	Canada	PAPVC, TGA, MAPCA, Fontan	MR (HoloLens)	Quantitative (n = 5)	Improved accuracy; reduced surgical time	Moderate
Schlegel et al. /2025 [26]	Germany	CHD	AR (HoloLens)	Quantitative (n = 128)	Strong interest in AR; need for implementation/training	Good
Tiwari et al., 2025 [25]	India	Complex CHD	VR (Meta Quest Pro)	Quantitative (n = 10)	Changed management decisions in 7/10 patients	Excellent

## Data Availability

No new data were created or analyzed in this study. Data sharing is not applicable.

## Supplementary Materials

The following supporting information can be downloaded at: https://www.mdpi.com/article/10.3390/healthcare14183086/s1, Table S1: Search strategy. Table S2: Summary of the selected articles. Table S3: Critical appraisal and Risk of bias.
