# Supplementary material for: Computer-Based Simulation Technologies in Pediatric Cardiovascular Diseases: A Systematic Review of Applications and Outcomes
Source: Healthcare (Basel). 2026 Sep 19;14(18):3086. doi: 10.3390/healthcare14183086 (PMC13606252; doi:10.3390/healthcare14183086)
Supplement: Supplementary file 1 [file healthcare-14-03086-s001.zip › Supplemantary Table S2 - Summary of reviewed articles.pdf]

Supplementary Table S2: Summary of the selected articles

No.	Authors/ year	Country	Objective	Methods	Disease/Application of computer-based simulation	Type of technology	Results
1	Tiwari et al. / 2025 [25]	India	To evaluate the role of VR in surgical decision-making for complex congenital heart diseases compared to conventional CT imaging	Quantitative Study (10 patients with complex CHD, including conditions like DORV, TGA, cardiac rhabdomyoma, PAPVC, TOF, and others)	CHD / 3D cardiac modeling and surgical planning	VR, Elucis™ software, and the Meta Quest Pro® headset	VR changed management decisions in 7 out of 10 patients. There was poor agreement between pre-VR and post-VR decisions (k = -0.21), indicating VR provided new insights.
2	Schlegel et al. /2025 [26]	Germany	To assess the needs and expectations of medical students and lecturers regarding the integration of AR into cardiovascular education at Heidelberg University	Quantitative Study (101 students and 27 lecturers focused on educational topics, a survey-based study with descriptive statistics and a Likert scale)	CHD, Coronary anomalies/ Teaching cardiovascular anatomy and interventions like transcatheter aortic valve replacement or ablation	Microsoft HoloLens 2; Unity and 3D Slicer for model segmentation and development	Both students and lecturers showed strong interest in AR-based teaching, particularly for visualizing complex cardiovascular topics like congenital heart defects. There was little prior experience with AR/VR, indicating a need for both implementation and training in medical education.
3	Ponzoni et al. / 2025 [27]	Canada	To explore the feasibility of MR for preoperative planning and intraoperative assistance in complex congenital heart surgery	Quantitative Study (for 5 patients with PAPVC, right atrial isomerism and dextrocardia, transposition of the great arteries with abnormal coronary pattern in situs inversus, MAPCAs, and failing fontan, MR is used for 3D holographic visualization, preoperative planning, and intraoperative guidance)	PAPVC, MAPCAs, Right atrial isomerism and Dextrocardia, failing Fontan / Interactive MR holographic models were used to simulate surgical approaches, such as redirecting anomalous veins, planning Fontan pathways, coronary reimplantation, unifocalization of MAPCAs, and virtual fit testing for total artificial heart implantation.	Microsoft HoloLens 2	MR provided an immersive, interactive 3D understanding of complex cardiac anatomy, allowing surgeons to simulate surgical approaches and optimize strategies before surgery. It provided real-time intraoperative guidance, improved accuracy and reduced surgical time. But the bulkiness of HoloLens 2 and software limitations, like the need for manual calibration and difficulty with mobile structures, currently restrict its routine intraoperative use.
4	Blonsky et al. / 2025 [28]	USA	To assess the impact of a VR educational curriculum on medical students' knowledge of CHD during their	Quantitative Study (373 medical students in different years of training studied six common CHDs)	CHDs, ASD, VSD, Aortic Stenosis, Pulmonic Stenosis, Patent Ductus Arteriosus, and Coarctation of the Aorta/ VR allowed users to be immersed	Stanford Virtual Heart software is Lighthaus Inc.; it was a self-directed, interactive VR	Medical students who used the VR curriculum scored significantly higher on a CHD knowledge assessment than the control group. Over 99% of participants

No.	Authors/ year	Country	Objective	Methods	Disease/Application of computer-based simulation	Type of technology	Results
			pediatric clerkship		inside a beating, interactive 3D model of a heart to explore normal anatomy and the six common CHDs listed above.	experience where users could examine, rotate, and expand 3D heart models with an integrated audio script and took approximately 30-45 minutes to complete.	recommended integrating the VR tool into the medical school curriculum. The VR experience was rated as easy to use and enjoyable by the vast majority (>92%) of students. A limitation was that some users (~28%) experienced motion sickness, though it did not prevent them from completing the tutorial.
5	Salavitabar et al./ 2024 [29]	USA	To evaluate the feasibility, image quality, and perceived benefits of converting 3D rotational angiography models into AR models for CHD visualization	Quantitative Study (30 CHD patients included truncus arteriosus, d-TGA s/p arterial switch operation, coarctation of the aorta, Single ventricle s/p Fontan, TOF variant, single ventricle s/p Hemi-Fontan/Glenn with a median age at catheterization of 6.5 years)	CHDs/ AR allowed interactive manipulation like scaling, slicing, and walking through models for enhanced spatial understanding, diagnostic and educational purposes.	Microsoft HoloLens 2 with AR headset, Canon Infinix-I fluoroscopic platform, and Vitrea 3D workstation (Vital Solutions, Canon) for 3D reconstruction	AR models were successfully created from 3D rotational angiography data with similar image quality to computer models. AR was rated very easy for visualizing structures in 81.1% of cases vs. 67.8% for computer models. AR provided superior 3D spatial understanding in most anatomies, except for aortic arch obstruction. AR was highly endorsed for use in patient or family counseling and trainee and staff education.
6	Mehta et al./ 2024 [30]	USA	To develop and evaluate a multi-user mobile AR application (ARCoLLAB) for collaborative cardiovascular surgical planning	Mixed-Methods study (A qualitative usability evaluation (with expert feedback and open-ended questions) and a quantitative component (a questionnaire using a 7-point Likert scale) for 5 participants, including 3 cardiothoracic surgeons and 2 cardiologists focused on CHDs)	CHDs / ARCoLLAB is a multi-user mobile AR iOS application designed for collaborative surgical planning, enabling users to interact with a 3D heart model generated from a patient's de-identified medical imaging data	ARCoLLAB was built natively for iOS using Apple's frameworks, including SwiftUI, RealityKit, ARKit, Multipeer Connectivity.	ARCoLLAB's multi-user collaborative features were highly rated for ease of use and were found to facilitate surgical planning. Anchoring the 3D model to a physical space was important for intuitive interaction from different perspectives. The portability of mobile AR and the familiarity of its gesture-based interface were crucial advantages over other XR technologies.
7	Jolley et al. /	USA	To describe the initial	Mixed-Methods study	Severe AVVR/ Virtual simulation	MitraClip G4 system,	TEER was successfully performed in

No.	Authors/ year	Country	Objective	Methods	Disease/Application of computer-based simulation	Type of technology	Results
	2024 [31]		experience and protocol for using the MitraClip system for transcatheter edge-to-edge repair (TEER) in congenital heart disease patients at a children's hospital	(four patients, two with hypoplastic left heart syndrome and fontan circulation, one with complete atrioventricular canal defect, and one with unbalanced complete atrioventricular canal and fontan circulation)	was performed using 3D Slicer and the SlicerHeart extension to model catheter trajectories, test access points, and optimize clip placement in a digital replica of the patient's anatomy.	3D Slicer, SlicerHeart, Tomtec Imaging Arena, Autodesk Fusion 360 were the software. Hardware included Philips EPIQ 7 ultrasound, Siemens 1.5T MRI, and Stratasys J750 3D printer.	all four high-risk CHD patients using a standardized imaging and simulation protocol. Significant reduction in AVVR was achieved in all cases, with improved hemodynamics and symptom relief. Ventricular function decreased acutely in some patients but recovered or improved at follow-up. The approach enabled safe and effective clip placement despite complex anatomies and limited prior experience.
8	Italiano et al./ 2023 [32]	Italy	To assess the feasibility and report the successful implantation of a HeartMate3 left ventricular assist device in a small child using a multimodality imaging and 3D simulation pathway	Quantitative study (10-year-old girl patient case report with end-stage heart failure due to MYH7-linked dilated cardiomyopathy)	HF/ CT-derived 3D reconstruction to create a virtual model of the chest cavity, simulating device implantation and determining the optimal position.	CT imaging and 3D reconstruction software, and the implanted device was the HeartMate3 (HM3) by Abbott	Preoperative 3D simulation successfully identified a feasible posterior position for the HeartMate3 pump in a child with a very small thoracic cavity. The device was implanted as planned and functioned excellently with normal parameters until transplantation. The simulation-guided approach was crucial for planning and can help expand the use of intracorporeal left ventricular assist device to smaller children.
9	Stepanenko et al./ 2023 [33]	Spain	To evaluate the use of 3D virtual modelling, printing, and extended reality for planning implant procedures of mechanical circulatory support devices and heart transplantation in complex adult heart	Quantitative Study (four patients with Ebstein anomaly (C type), calcified septo-apical left ventricular aneurysm, left ventricular aneurysm with extensive endocardial calcification, and mitral-aortic dehiscence with	Ebstein anomaly (C type), Calcified septo-apical left ventricular aneurysm, Left ventricular aneurysm with extensive endocardial calcification, Mitro-aortic dehiscence with pseudoaneurysm after previous endocarditis and surgery/	Mimics Innovation Suite (Materialise), Rhinoceros 3D software, and Formlabs Form 3 SLA 3D printer as hardware. Microsoft HoloLens AR and VR. Devices modelled by	3D virtual and printed models improved surgical planning and changed the treatment approach in complex heart failure cases. They enabled successful implantation of ventricular assist devices in challenging anatomies by optimizing cannula placement and avoiding calcified tissues. In high-risk cases,

No.	Authors/ year	Country	Objective	Methods	Disease/Application of computer-based simulation	Type of technology	Results
			failure patients	pseudoaneurysm after previous endocarditis and surgery).	Preoperative planning, virtual device implantation, and surgical simulation to optimize device positioning and surgical approaches	Impella RP (Abiomed) and HeartMate 3 (Abbott).	technology helped redirect treatment toward heart transplantation. Extended reality and 3D printing enhanced spatial understanding, procedural confidence, and team preparedness.
10	Peek et al./ 2023 [34]	Netherlands	To assess the added value of 3D printing and 3D VR over conventional 2D imaging for surgical planning in patients with DORV	Quantitative Study (five patients with DORV and 12 participants (five congenital cardiothoracic surgeons and seven pediatric cardiologists)/ proposed surgical plans using 3D VR for immersive, interactive visualization of cardiac anatomy and surgical planning)	DORV/ Segmented DICOM CT data were converted into 3D-VR models using MedicalVR software, allowing users to interact with the model using VR controllers and virtual cutting planes.	The software is 3D Slicer and MedicalVR (Amsterdam, Netherlands,) and the MedicalVR workstation is the hardware.	3D VR and 3D printing provided better visualization of spatial relationships than 2D imaging. Surgical plans based on 3D VR and 3D printing more closely matched the actual performed surgery (80% and 78% vs. 66% for 2D). 3D VR was particularly effective for assessing VSD patch closure feasibility (92% vs. 66% for 3D print and 46% for 2D). Both 3D methods were considered useful supplements to conventional imaging, but not replacements for it.
11	Miyagi et al./ 2023 [35]	USA	To determine the anatomical fit of two small-sized (pediatric and infant) continuous-flow total artificial heart pumps in CHD patients	Quantitative Study (40 patients with CHD; some also had dilated cardiomyopathy)	CHD and dilated cardiomyopathy/ Preoperative CT scans were converted into 3D models, and virtual pump models were placed within these anatomical models to assess fit and interference with surrounding tissues.	Mimics Medical (Materialise), SOLIDWORKS (Dassault Systèmes), and 3D-printed physical models of the pumps	The pediatric continuous-flow total artificial heart pumps fit (52.5%) and the infant continuous-flow total artificial heart pumps fit (100% of 33 patients) were evaluated. Preoperative X-ray measurements were useful predictors of pump fit. Virtual 3D CT fitting confirmed visual and radiographic findings, supporting feasibility in small pediatric patients.
12	Lim et al./ 2023 [36]	USA	To evaluate the efficacy of a virtual reality curriculum in increasing pediatric residents'	Quantitative Study (142 participants (80 control, 62 intervention), and CHDs covered were ASD, VSD,	CHDs/ To explore anatomy, follow blood flow, and learn about common CHDs via guided audio modules.	VR Lighthaus Inc.	Trainees who used the VR curriculum scored significantly higher on a validated assessment than the control group. The

No.	Authors/ year	Country	Objective	Methods	Disease/Application of computer-based simulation	Type of technology	Results
			knowledge of CHDs	aortic stenosis, pulmonic stenosis, coarctation of the aorta, and patent ductus arteriosus. The largest percentage of learners (45.8%) were postgraduate year 1).			improvement was most notable on questions testing visuospatial concepts, such as chamber dilation from shunts. User feedback was overwhelmingly positive, with all respondents recommending its integration into the residency curriculum. VR is an effective and engaging educational tool for teaching complex cardiac anatomy and physiology.
13	Bertelli et al./ 2023 [37]	Italy	To validate the use of DIVA software for fast and accurate 3D reconstruction of congenital heart defects by inexperienced users	Quantitative Study (Five patients with right partial anomalous pulmonary venous return and five participants (2 medical students and 3 cardiac surgery residents))	Right partial anomalous pulmonary venous return/ Internal navigation and manipulation of anatomical structures	DIVA software was developed at Institut Pasteur, Paris, and built with the Unity game engine.	Inexperienced users produced accurate 3D reconstructions with minimal training. Quality and speed improved significantly across five cases. DIVA allows fast-track 3D modeling suitable for clinical use. VR-enhanced models provided superior spatial understanding compared to 2D images.
14	Awori et al./ 2023 [38]	USA	To compare the perceived educational effectiveness of VR versus 3D printed models in teaching cardiac anatomy and pathophysiology to pediatric residents and nurse practitioners	Quantitative Study (30 participants (27 pediatric residents, 3 nurse practitioners)	Developmentally typical heart, CHD, TOF/ To manipulate a virtual slicing tool within the VR environment to view heart models from multiple planes.	VR/AR system with a stereoscopic display, stylus, polarized glasses, and Z-Space software, version 5.0 (zCORE® San Jose, CA)	Participants reported significantly higher understanding with VR models (8.5/10) compared to 3D printed models (6.3/10) and traditional methods (5.5/10). 87% of participants preferred VR over 3D printed models. VR was praised for its multi-planar visualization capabilities, while 3D printed models were limited by fixed cutting planes.
15	Ghosh et al. / 2022 [9]	USA	To describe the growth, development, and integration of a clinical 3D modeling service for procedural planning in	Quantitative Study (112 individual 3D modeling cases with CHD and other complex cardiac pathologies performed at	CHD / VR as one of the 3D visualization modalities in the clinical 3D modeling service for pediatric cardiothoracic surgery	VR through the Slicer VR module in 3D Slicer software, Valve Index Headset, and VR were used for	The clinical application of 3D modeling tools, including 3D printed models, digital 3D models, CAD-based modeling, and VR, can be seamlessly integrated into pre-

No.	Authors/ year	Country	Objective	Methods	Disease/Application of computer-based simulation	Type of technology	Results
			pediatric cardiothoracic surgery and intervention	The Children's Hospital of Philadelphia from 2018 to 2020)		virtual interaction with 3D models.	procedural care for pediatric patients with CHD. 3D printed models offer tactile and interactive insights into complex anatomy, digital models enable intuitive visualization, CAD-based modeling facilitates precise surgical design, and VR immerses clinicians in virtual patient anatomy.
16	Vegulla et al./ 2022 [12]	USA	To explore the use of advanced cardiac imaging modalities (CT and MRI) and VR simulation to digitize and assess the feasibility of imaging preserved heart specimens with CHD for educational and research purposes	Quantitative Study (7 Specimen of a specific group of patients with CHD Pathology of Specimen 1 indicates DORV, ASD, VSD, Patent ductus arteriosus, and post-Fontan surgery. Pathology of Specimen 2 indicates TOF. Pathology of Specimen 3 indicates pulmonary atresia, hypoplastic right ventricle, duct, and atrial septectomy performed. Specimen 4 is a normal heart. Pathology of Specimen 5 indicates D-TGA, pulmonary and tricuspid atresia, post-Fontan surgery. Pathology of Specimen 6 indicates Perimembranous VSD. Pathology of Specimen 7 indicates D-TGA.)	CHD / VR was used to visualize and interact with the 3D virtual models of preserved heart specimens	HTC Vive Headset and VR-capable computer created an immersive virtual environment to explore digitized heart models.	The 3D virtual models in VR space allowed for intuitive exploration and understanding of complex cardiac morphology and congenital heart defects and anatomy. Using VR for immersive exploration emphasizes VR's advantage in interactive learning over traditional handling of physical specimens and enhances cardiac education for medical professionals and students. Making cMRI a more suitable method for digitizing and studying preserved heart specimens in VR.
17	Ye et al. /	China	To assess the feasibility	Quantitative Study (34	DORV/ Radiology-surgical	MR holographic	The study contrasts the use of

No.	Authors/ year	Country	Objective	Methods	Disease/Application of computer-based simulation	Type of technology	Results
	2021 [39]		and accuracy of using MR technology in the diagnosis and surgical planning of patients with DORV	cardiac CT angiography scans of patients suspected of DORV based on ultrasound findings)	planning	visualization	MRHG and conventional methods for diagnosing and planning surgeries for DORV. MRHG achieved 100% accuracy in DORV type diagnosis, compared to 88.2% in the control group. Accompanying malformation diagnosis accuracy was 95.5% in MRHG and 89.7% in the control. MRHG showed shorter surgical planning times for DORV diagnoses and procedures. The DORV type diagnosis did not significantly differ between groups, and the MRHG group saw no surgical changes, in contrast to three modifications in the control group.
18	Vigil et al./ 2021 [40]	USA	To describe a newly developed methodology for creating virtual baffles to inform intraoperative repair of DORV	Quantitative Study (10 months patient with DORV, Sub-Aortic VSD, Pulmonary Stenosis - 7 months patient with DORV, Sub-Aortic VSD, Prominent Conus - 6 months patient with DORV, Valvar Pulmonic Stenosis, and Sub-Pulmonary VSD)	DORV / VR was used in the context of preoperative planning for the repair of DORV using virtual baffles	VR, HTC Vive Headset, and SlicerVR to visualize the resulting models, and to enhance the visualization and understanding of the complex intracardiac anatomy and baffle pathways.	The newly developed baffle planning tool enabled simpler and faster interaction and refinement of 3D baffles, compared to traditional segmentation-based tools. This streamlined preoperative planning for complex DORV repairs. Moreover, the paper introduces a novel method for generating physical templates from virtual baffle models using 3D printing and flattening.
19	van de Woestijne et al. / 2021 [41]	Netherlands	To assess the technical feasibility, clinical usefulness, and user experience of 3D-VR imaging for preoperative planning in newborns with	Quantitative Study (7 patients with pulmonary atresia and MAPCAs underwent corrective repair between 2010 and 2020. These patients were included in the	Pulmonary atresia and MAPCAs / 3D-VR used as a complementary modality for preoperative planning of MAPCA repair in CHD patients	VR application was referred to as CardioVR, and it was developed by MedicalVR and included 3D-VR visualization with VR	The use of 3D-VR in preoperative planning showed additional value in identifying specific anatomical characteristics, potentially influencing surgical decisions, and improving understanding of their complex anatomy and surgical

No.	Authors/ year	Country	Objective	Methods	Disease/Application of computer-based simulation	Type of technology	Results
			pulmonary atresia and MAPCAs	retrospective proof-of-concept study to assess the feasibility and clinical usefulness of 3D-VR imaging for preoperative planning in newborns with pulmonary atresia and MAPCAs)		HMD and associated controllers	outcomes. The surgeons rated the VR environment as easy to use and found additional value in assessing MAPCA characteristics, suggesting potential benefits for enhancing surgical procedures.
20	Raimondi et al. / 2021 [8]	France	To evaluate the appropriateness of using a new VR technology directly applied to raw MRI data for rendering cardiac anatomy in comparison to standard 3D rendering techniques (3D PDF and 3D printing) in patients with complex CHD	Quantitative Study (Case 1 with dextrocardia, and partial anomalous pulmonary venous drainage of the middle and lower lobe of the right lung into the inferior vena cava (scimitar syndrome), Case 2 with ventricular D-loop, DORV with hypoplasia of the left ventricle, hypoplasia of the mitral valve, pulmonary valve atresia, and subaortic VSD, Case 3 with ventricular D-loop, atrioventricular concordance, ventriculoatrial concordance with normally positioned aorta)	Different types of CHD/ VR was used for visualizing and comprehending intracardiac and vascular anatomy in complex CHDs	A VR tool named DIVA (Directly Integrated VR Application), was custom-built for this research using the Unity game engine. DIVA offers dual interfaces: desktop mode for standard monitors and VR mode for headset-based medical image interaction.	The VR showcases improved visualization (Cases 2 and 3), expedited processing (5 minutes vs. 8 hours for 3D), and implicit benefits, signifying its transformative potential in reshaping 3D modeling. The results highlighted the potential of the newly introduced VR method, directly applied to raw MRI data, to revolutionize advanced 3D modeling for complex CHD, ensuring enhanced anatomical insights and considerably swifter processing compared to traditional techniques.
21	Milano et al. / 2021 [42]	United Kingdom	To compare the effectiveness of various 3D modeling techniques, including 3D pdf, 3D printed models, and VR, in the	Quantitative Study (10 patients with Complex DORV and complex interventricular communications)	Complex DORV / VR 3D modeling modality in planning complex congenital heart surgeries for patients with DORV	VR Oculus Rift system consists of a VR headset, two sensors, and two hand controllers for grabbing, cutting,	The results showed highest accuracy with VR in identifying suitable candidates and need for arterial switch. The use of VR increased the concordance between surgeon decisions and actual surgical plans,

No.	Authors/ year	Country	Objective	Methods	Disease/Application of computer-based simulation	Type of technology	Results
			preoperative planning of complex congenital heart surgeries for patients with DORV			measuring, marking, multiplanar reconstruction, and virtual eco tools	providing a more immersive and intuitive way to assess patient-specific anatomy. VR improved the identification of ideal candidates for biventricular repair to 95% and the need for arterial switch operation within biventricular repair to 50%.
22	Pushparajah et al. / 2021 [43]	United Kingdom	To investigate the impact of VR imaging on decision-making in atrioventricular valve surgery in pediatric patients with CHD	Quantitative Study (15 pediatrics who required surgical repair of atrioventricular valves)	CHD/ VR for visualizing 3D echocardiographic data for surgical planning in atrioventricular valve repair	VR application developed in-house using Unity, Visualization Toolkit, and utilized with an HTC Vive VR Headset, offered dynamic 3D images, immersive visualization, anatomic landmarks, and orientation for surgical planning	Using VR imaging in surgical planning for atrioventricular valve repair in CHD improves surgeons' understanding and confidence in the pathology, leading to potential modifications in the surgical approach in the majority of cases. The main benefit of VR is the enhanced visualization of anatomical structures and dynamic images of the valve apparatus, providing valuable insights for surgical decision-making.
23	Gehrsitz et al./ 2021 [44]	Germany	To assess the benefits of using Cinematic Rendering-holograms in the preoperative planning of pediatric heart surgery and compare them with 3D printing and 2D imaging methods	Quantitative Study (26 patients with CHD)	CHD/ MR Preoperative planning for pediatric heart surgery	Cinematic rendering holograms from the HoloLens® mixed-reality technology, combined with the realistic cinematic rendering of the patient's heart	The mixed-reality visualization imaging software "syngo.via" application, which generated cinematically rendered holograms (CR-holograms) of pediatric patients' hearts, was superior to traditional 2D screen imaging and 3D-printed models in preoperative planning for heart surgery. The use of CR-holograms led to improved depth perception, better representation of pathology, and a significant reduction in intraoperative preparation time, making it a promising tool for enhancing

No.	Authors/ year	Country	Objective	Methods	Disease/Application of computer-based simulation	Type of technology	Results
							surgical outcomes in pediatric cardiovascular procedures.
24	Huang et al. / 2021 [45]	USA	To introduce and describe the ongoing work of developing the Phoenix Virtual Heart names PVH, a hybrid VR-desktop software, to aid in CHD surgical planning and family consultation, integrated into a 3D printing workflow at a children's hospital	Qualitative Study (no information about patients or participants)	CHDs / PVH was a hybrid VR-desktop software developed to aid in surgical planning and family consultation for complex, structural CHDs cases	PVH VR for an intuitive and immersive platform for physicians to analyze complex CHD cases, enhance surgical planning and interventions, improve family consultation, spatial insight into the patient's heart anatomy, simulate implantation scenarios, and assess their fit and plan for procedures	By integrating the strengths of both VR and desktop interactions, PVH offered improved 3D intelligence, realistic depth perception, and effective modalities for CHD analysis and decision-making. While 3D printed models offer benefits like better understanding of complex anatomy, their limited annotation capacity prompts physicians to favor software-based planning. PVH has garnered positive feedback from physicians.
25	Cen et al. / 2021 [46]	China	To evaluate the surgical outcomes of patients with pulmonary atresia and VSD with major aortopulmonary collateral arteries (MAPCA) after using a combination of 3D-printed heart models, VR, and MR in guiding surgical planning	Quantitative Study (five patients had the combination of PA, VSD, and MAPCA and Age 5.86 ± 1.2 years in mean)	Pulmonary atresia, VSD, and MAPCA / VR for surgical planning processes	VR technology with VR glasses for immersive analysis of complex cardiac anatomy and surgical planning, MR provided holographic visualization	The preoperative use of 3D-printed heart models with VR or MR significantly improved the surgeons' understanding of intricate cardiac anatomy, resulting in satisfactory surgical outcomes for patients with PA and VSD with MAPCA. 3D-printed models enhance tactile understanding of complex cardiac anatomy, aiding preoperative planning. The combination of 3D-printed heart models, VR, and MR proved beneficial in guiding surgical planning for Pulmonary atresia and VSD patients
26	Priya et al./	USA	To demonstrate a	Quantitative Study (5-	TOF / VR and compatible mixed-	VR visualization	This paper delves into a comparison

No.	Authors/ year	Country	Objective	Methods	Disease/Application of computer-based simulation	Type of technology	Results
	2020 [47]		proof-of-concept study for creating a 4-D cine model of the heart using open-source software and visualizing it in VR, aiming to improve understanding of cardiac anatomy and potentially aid in patient and trainee education as well as surgical planning	month-old boy with TOF)	reality glasses provide an efficient and immersive way to explore and understand complex CHD in 4-D space	utilized mixed-reality glasses (HP Windows mixed-reality headset) and handheld motion controllers, supported by Slicer software, enabling clear spatial insights, dynamic 4-D cine cardiac models, and interactive manipulation	between 3D printed models and VR four-dimensional (4D) models to enhance cardiac anatomy visualization. While 3D prints lack motion and real-time adaptability, VR 4D models introduce dynamic interaction, adjustable cutting planes, and cost-effectiveness, revolutionizing medical education, simulation, and communication.
27	Capellini et al. / 2020 [48]	Italy	To test and compare the accuracy and incremental diagnostic value of 3D printed and virtual models in patients undergoing cardiac surgery for congenital heart diseases (CHDs)	Quantitative Study (12 Magnetic Resonance (MR) and 8 CT volumetric datasets)	CHDs / VR was utilized to provide an immersive, interactive experience for cardiac surgeons and pediatric cardiologists to visualize and plan interventions using 3D models of patients' hearts and cardiac structures in CHD cases	VR platform built with Unity engine and Oculus Rift HMD for compatibility, enabled visualization and planning of patient-specific cardiac structures, surgical and percutaneous planning for patients with complex CHDs	A combination of 3D printed and virtual models enhanced surgical and percutaneous planning for patients with CHDs, enabling clinicians to explore multiple access sites and refine intervention strategies. The VR technology provided an immersive environment for manipulating and visualizing 3D heart models, improving decision-making and confidence in surgical approaches.
28	Kang et al. / 2020 [49]	Canada	To evaluate the accuracy and utility of a mixed-reality system in the morphological assessment of complex CHD, specifically Fallot-type DORV, with correlation to anatomical specimens	Quantitative Study (12 heart specimens with DORV pathology)	DORV / To interrogate the 3D stereoscopic images of the cardiac specimens and for a better understanding of the spatially complex lesions in Fallot-type DORV	Mixed-reality system named True 3D from Echopixel (The system provided an interactive stereoscopic experience through the augmentation of binocular parallax motion and	The findings demonstrated that a mixed-reality system provided highly accurate and realistic stereoscopic representations of spatially complex congenital cardiac lesions, enhancing 3D understanding of morphology and spatial relationships in the Fallot-type DORV. The research affirms the viability of employing clinical CT

No.	Authors/ year	Country	Objective	Methods	Disease/Application of computer-based simulation	Type of technology	Results
						kinesthetic feedback. It allowed for the creation of computer-generated stereoscopic images from CT imaging data of the cardiac specimens.)	scanners to digitize cardiac specimens, serving the dual goals of long-term preservation and educational applications.
29	Brun et al. / 2019 [50]	Norway	To establish proof of concept and examine the feasibility and initial user experience of a new MR hologram platform for diagnosis and treatment planning in complex CHD	Quantitative Study (a 9-year-old boy patient with DORV and transposition of the great arteries (DORV-TGA))	DORV-TGA / Using MR hologram application to diagnose and plan surgery for complex CHD, specifically in the case of a patient with DORV-TGA	MR application with the Microsoft HoloLens® platform and a wearable MR viewing device	The MR hologram platform, utilizing the Microsoft HoloLens®, effectively assists in diagnosing complex CHD and aids in surgical planning, as demonstrated by accurate identification of anatomical structures, correct diagnosis, and alignment with surgical decisions. Participants reported highly positive user experiences with the hologram's 3D depth perception and morphology understanding, along with favorable ratings for manipulation tools, especially among younger and female professionals.
30	Ong et al. / 2018 [51]	USA	To demonstrate the novel use of VR for the presurgical planning of cardiac surgery in complex CHD faces	Quantitative Study (A 2.95 kg baby girl with Truncus Arteriosus type A4 with ascending aortic arch hypoplasia and a 5-month-old (35-week premature infant) with Large VSD and right-sided congenital diaphragmatic hernia)	Two specific cases of complex CHD / VR-assisted presurgical planning	HTC[1] Vive VR headset with two controllers for interactive and shared viewing, manipulation and collaboration of the 3D heart models	The findings showed that VR is feasible and effective for presurgical planning in complex CHD cases, providing interactive real-time views of patient-specific anatomy and serving as a useful complement to traditional imaging modalities and 3D printed heart models. The paper compares VR technology

[1] High Tech Computer Corporation (Taiwanese company)

No.	Authors/ year	Country	Objective	Methods	Disease/Application of computer-based simulation	Type of technology	Results
							for presurgical planning with 2D visualizations and 3D-printed heart models. The comparisons underscore VR's advantages in providing unique insights, adjustable views, and improved multidisciplinary communication,
31	Olivieri et al. / 2018 [7]	USA	To evaluate the impact of patient-specific virtual 3D heart models on pediatric cardiac intensive care unit provider attitudes and care delivery	Quantitative Study (20 cases with different cardiac diseases such as LPA sling, total anomalous pulmonary venous connection, ASD, right arch with aberrant left subclavian, kommerell diverticulum,TOF, etc.)	CHDs / Training for healthcare providers in critical care units for patients with congenital heart defects (CHDs)	Asynchronous nature of the simulation Virtual 3D heart models using the Cardiac Review 3D software on tablets	Using simulation-based methods with virtual 3D heart models can have a positive impact on healthcare providers' understanding, confidence, and care delivery for patients with congenital heart defects in critical care. Compared to the traditional methods, interacting with virtual 3D models led to enhanced comprehension of anatomy, improved ability to manage postoperative complications, and increased confidence in handoffs. Additionally, a cost analysis indicated that virtual 3D models offered cost savings while maintaining significant value in improving patient care.
32	Pereira et al. / 2016 [52]	Portugal	To assess the pedagogical impact of using virtual patient simulation technology in a short workshop format for training medical students to detect cardiac pathologies through auscultation	Quantitative Study (A total of 117 medical students (third year: 75, fourth year: 26, fifth-year: 16) participated in eleven two-hour cardiac auscultation workshops using electronic stethoscopes in a clinical setting.	Cardiac pathologies like Aortic Stenosis, Pulmonic stenosis, Aortic Regurgitation, Mitral Regurgitation, and VSD/To increase medical students' exposure to varied auscultation findings and enhance training hours.	IS4Learning Web was selected due to its compatibility with web platforms, facilitating integration.	The findings revealed a significant improvement (+16%) in differentiating between normal and pathological heart sounds after a short workshop using virtual patient simulation technology. However, the complexity of distinguishing specific pathologies, such as Aortic Regurgitation and Mitral Regurgitation, showed limited

No.	Authors/ year	Country	Objective	Methods	Disease/Application of computer-based simulation	Type of technology	Results
							improvement. The research highlighted the potential of virtual simulators for enhancing auscultation skills and building confidence among medical students.
33	Rao et al. / 2015 [53]	USA	To establish an image-based morphometry paradigm for assisting in virtual surgical planning and functional evaluation in patients with chronic TOF and severe LPA stenosis	Quantitative Study (2 specific cases of patients with chronic ToF and severe LPA)	Chronic ToF and LPA / Virtual simulation offers insights into diverse scenarios, supporting optimized surgical choices for individual patients while complementing, rather than replacing, actual surgical procedures.	Virtual simulation for patients' digital anatomical models, simulate surgical steps, assess hemodynamics and pressure gradients, surgical decision-making by predicting outcomes	Virtual simulation as a pivotal tool for refining surgical planning and decision-making demonstrated the capacity of analyzing diverse surgical scenarios, systematically evaluating potential outcomes, and foreseeing the repercussions of various interventions in a controlled and predictive framework. The virtual simulation-guided surgical approach effectively improved hemodynamics in pulmonary arteries for both LPA and Right Ventricular Outflow Tract stenosis cases, as evidenced by decreased pressure gradients.
34	De Zélicourt et al. / 2011 [54]	USA	To use virtual surgery and simulations to identify optimal surgical strategies for patients with a single ventricle and interrupted inferior vena cava, aiming to address pulmonary arteriovenous malformations caused by abnormal hepatic flow distribution	Quantitative Study (5 patients who had a single ventricle and an interrupted inferior vena cava)	Pulmonary arteriovenous malformations caused by abnormal hepatic flow distribution / Virtual simulation process allowed the researchers to assess a range of surgical scenarios without physically intervening in patients and helped identify potential surgical strategies that would lead to improved hepatic flow distribution and decreased risk of pulmonary arteriovenous malformations.	Novel virtual surgery interface simulation to create virtual simulations and generate various corrective anatomies	Precise placement of hepatic baffles through various surgical options improved oxygen saturation and reduced PAVM risk. Strategies like Y-grafts and hepato-to-azygous shunts showed promise for single SVC cases, while mid-PA connections proved effective for bilateral SVC cases. Novel virtual surgery simulations aided in identifying effective corrective approaches based on available techniques and individual anatomical constraints.
35	Xue et al. /	China	To introduce and	Quantitative Study (10	ASD, VSD, TOF, DORV, AVSD /	3D	Virtual endoscopy using 3DE IESS

No.	Authors/ year	Country	Objective	Methods	Disease/Application of computer-based simulation	Type of technology	Results
	2010 [55]		analyze the feasibility and accuracy of a novel visualization method called the "3D echocardiographic intracardiac endoscopic simulation system (3DE IESS)" using virtual endoscopy for diagnosing CHD in children	Normal subjects, 9 patients with ASD, 4 patients with VSD, 4 patients with TOF, 2 patients with both ASD and VSD, 4 patients with both TOF and ASD, 5 patients with both DORV and VSD, 1 patient with both DORV and AVSD, 1 patient with DORV, VSD, and ASD were included)	Virtual endoscopy for diagnosing CHD in children	echocardiographic intracardiac endoscopic simulation system (3DE IESS)"	achieved a diagnostic accuracy of 98.7% for ASD, 92.4% for VSD, 92.6% for TOF, and 94% for DORV. Virtual endoscopy showed significantly higher diagnostic accuracy compared to two-dimensional echocardiography for TOF and DORV, indicating its potential as a valuable tool for diagnosing CHD and enhancing the understanding of intracardiac structures.
36	Riesenkampff et al. / 2009 [56]	Germany	To assess the practical clinical value of realistic 3-dimensional models of complex congenitally malformed hearts for surgical planning and decision-making	Quantitative Study (11 patients with complex congenitally malformed hearts include various congenital heart defects such as DORV, TOF, congenitally corrected transposition, AVSD, and more)	Complex congenitally malformed hearts / Virtual simulation offered an intricate and detailed representation of the intracardiac anatomy, empowering surgeons and cardiologists to enhance their comprehension of intricate pathology, arrive at more informed choices regarding surgical methodologies, and anticipate potential results of surgical interventions	Virtual 3D computer models of complex congenitally malformed hearts based on high-resolution whole-heart or cine MRI and CT data	The virtual 3D models significantly enhanced surgical decision-making for intricate congenital heart conditions by providing precise anatomical insights and faithful representations of intraoperative discoveries. They effectively guided surgical recommendations and fostered consensus among medical experts while elevating preoperative planning. These models empowered healthcare professionals to make better-informed decisions about treatment strategies for patients with intricate congenital heart conditions, ultimately leading to more effective therapeutic approaches and improved patient outcomes.

Table S2 Abbreviations: 3D: Three-Dimensional; 3D-VR: Three-Dimensional Virtual Reality; 3DE IESS: Three-Dimensional Echocardiographic Intracardiac Endoscopic Simulation System; ASD: Atrial Septal Defect; AVSD: Atrioventricular Septal Defect; AVVR: Atrioventricular Valve Regurgitation; CAD: Computer-Aided Design; CHD: Congenital Heart Disease; CR-holograms: Cinematically Rendered Holograms; CT: Computed

Tomography; CMR: Cardiac Magnetic Resonance; DIVA: Directly Integrated Virtual Reality Application; DICOM: Digital Imaging and Communications in Medicine; DORV: Double-Outlet Right Ventricle; D-TGA: Dextro-Transposition of the Great Arteries; HMD: Head-Mounted Display; LPA: Left Pulmonary Artery; MAPCAs: Major Aortopulmonary Collateral Arteries; MR: Mixed Reality; MRHG: Mixed-Reality Holographic Guidance; MRI: Magnetic Resonance Imaging; PAPVC: Partial anomalous pulmonary venous connection; PVH: Phoenix Virtual Heart; SVC: Superior Vena Cava; TGA: Transposition of the Great Arteries; TOF: Tetralogy of Fallot; VSD: Ventricular Septal Defect; VR: Virtual Reality.
