# Supplementary material for: Computer-Based Simulation Technologies in Pediatric Cardiovascular Diseases: A Systematic Review of Applications and Outcomes
Source: Healthcare (Basel). 2026 Sep 19;14(18):3086. doi: 10.3390/healthcare14183086 (PMC13606252; doi:10.3390/healthcare14183086)
Supplement: Supplementary file 1 [file healthcare-14-03086-s001.zip › Supplementary Table S1 - Search Strategy.pdf]

Supplementary Table S1: Search strategy

Database name	Search strategy
**PubMed**	(("Computer Simulation*"[mesh] OR "Augmented Reality"[mesh] OR "Patient-Specific Modeling"[mesh] OR "Virtual Reality"[mesh] OR "Patient Simulation*"[mesh] OR "Mixed Reality"[Title/Abstract] OR "Educational Virtual Reality"[Title/Abstract] OR "Instructional Virtual Reality"[Title/Abstract] OR "Patient-Specific Computational Modeling"[Title/Abstract] OR "Interactive Learning"[Title/Abstract]) AND ("Cardiovascular Disease*"[mesh] OR "Heart Disease*"[mesh] OR "Heart Disorder*"[Title/Abstract] OR "Cardiac Disease*"[Title/Abstract] OR "Cardiac Disorder*"[Title/Abstract] OR "Heart Defect*"[Title/Abstract]) AND ("Pediatric*"[mesh] OR "Child"[mesh] OR "Children"[Title/Abstract])) AND (english[Language]) AND ("2025/09/01"[Date - Publication] : "2025/09/01"[Date - Publication])
**Ovid**	((("Computer Simulation*" or "Augmented Reality" or "Patient-Specific Modeling" or "Virtual Reality" or "Patient Simulation*" or "Mixed Reality" or "Educational Virtual Reality" or "Instructional Virtual Reality" or "Patient-Specific Computational Modeling" or "Interactive Learning") and ("Cardiovascular Disease*" or "Heart Disease*" or "Heart Disorder*" or "Cardiac Disease*" or "Cardiac Disorder*" or "Heart Defect*") and ("Pediatric*" or "Child" or "Children")).ab,kw,ti.) AND (english.lg.) AND (up to 2025/09/01)
**Web of Science**	(((TI=((("Computer Simulation*" OR "Augmented Reality" OR "Patient-Specific Modeling" OR "Virtual Reality" OR "Patient Simulation*" OR "Mixed Reality" OR "Educational Virtual Reality" OR "Instructional Virtual Reality" OR "Patient-Specific Computational Modeling" OR "Interactive Learning") AND ("Cardiovascular Disease*" OR "Heart Disease*" OR "Heart Disorder*" OR "Cardiac Disease*" OR "Cardiac Disorder*" OR "Heart Defect*") AND ("Pediatric*" OR "Child" OR "Children")))) OR AB=((("Computer Simulation*" OR "Augmented Reality" OR "Patient-Specific Modeling" OR "Virtual Reality" OR "Patient Simulation*" OR "Mixed Reality" OR "Educational Virtual Reality" OR "Instructional Virtual Reality" OR "Patient-Specific Computational Modeling" OR "Interactive Learning") AND ("Cardiovascular Disease*" OR "Heart Disease*" OR "Heart Disorder*" OR "Cardiac Disease*" OR "Cardiac Disorder*" OR "Heart Defect*") AND ("Pediatric*" OR "Child" OR "Children")))) OR KP=((("Computer Simulation*" OR "Augmented Reality" OR "Patient-Specific Modeling" OR "Virtual Reality" OR "Patient Simulation*" OR "Mixed Reality" OR "Educational Virtual Reality" OR "Instructional Virtual Reality" OR "Patient-Specific Computational Modeling" OR "Interactive Learning") AND ("Cardiovascular Disease*" OR "Heart Disease*" OR "Heart Disorder*" OR "Cardiac Disease*" OR "Cardiac Disorder*" OR "Heart Defect*") AND ("Pediatric*" OR "Child" OR "Children")))) OR AK=((("Computer Simulation*" OR "Augmented Reality" OR "Patient-Specific Modeling" OR "Virtual Reality" OR "Patient Simulation*" OR "Mixed Reality" OR "Educational Virtual Reality" OR "Instructional Virtual Reality" OR "Patient-Specific Computational Modeling" OR "Interactive Learning") AND ("Cardiovascular Disease*" OR "Heart Disease*" OR "Heart Disorder*" OR "Cardiac Disease*" OR "Cardiac Disorder*" OR "Heart Defect*") AND ("Pediatric*" OR "Child" OR "Children"))) AND English (Languages) AND Publication Date: up to 2025-09-01
**IEEE Xplore**	(("Computer Simulation*" OR "Augmented Reality" OR "Patient-Specific Modeling" OR "Virtual Reality" OR "Patient Simulation*" OR "Mixed Reality" OR "Educational Virtual Reality" OR "Instructional Virtual Reality" OR "Patient-Specific Computational Modeling" OR "Interactive Learning") AND ("Cardiovascular Disease*" OR "Heart Disease*" OR "Heart Disorder*" OR "Cardiac Disease*" OR "Cardiac Disorder*" OR "Heart Defect") AND ("Pediatric*" OR "Child" OR "Children")) AND Filters: English, Year: ≤2025
**Scopus**	TITLE-ABS-KEY((("Computer Simulation*" OR "Augmented Reality" OR "Patient-Specific Modeling" OR "Virtual Reality" OR "Patient Simulation*" OR "Mixed Reality" OR "Educational Virtual Reality" OR "Instructional Virtual Reality" OR "Patient-Specific Computational Modeling" OR "Interactive Learning") AND ("Cardiovascular Disease*" OR "Heart Disease*" OR "Heart Disorder*" OR "Cardiac Disease*" OR "Cardiac Disorder*" OR "Heart Defect*") AND ("Pediatric*" OR "Child" OR "Children"))) AND (LIMIT-TO(LANGUAGE, "English")) AND (PUBYEAR ≤ 2025)
**Cochrane**	(((("Computer Simulation*" OR "Augmented Reality" OR "Patient-Specific Modeling" OR "Virtual Reality" OR "Patient Simulation*" OR "Mixed Reality" OR "Educational Virtual Reality" OR

Database name	Search strategy
	"Instructional Virtual Reality" OR "Patient-Specific Computational Modeling" OR "Interactive Learning") AND ("Cardiovascular Disease*" OR "Heart Disease*" OR "Heart Disorder*" OR "Cardiac Disease*" OR "Cardiac Disorder*" OR "Heart Defect*") AND ("Pediatric*" OR "Child" OR "Children"))):ti,ab,kw) AND (Language: English) AND (Date: up to 2025/09/01)
**EBSCO Host - Academic Search Premier**	TI ((("Computer Simulation*" OR "Augmented Reality" OR "Patient-Specific Modeling" OR "Virtual Reality" OR "Patient Simulation*" OR "Mixed Reality" OR "Educational Virtual Reality" OR "Instructional Virtual Reality" OR "Patient-Specific Computational Modeling" OR "Interactive Learning") AND ("Cardiovascular Disease*" OR "Heart Disease*" OR "Heart Disorder*" OR "Cardiac Disease*" OR "Cardiac Disorder*" OR "Heart Defect*") AND ("Pediatric*" OR "Child" OR "Children"))) OR AB ((("Computer Simulation*" OR "Augmented Reality" OR "Patient-Specific Modeling" OR "Virtual Reality" OR "Patient Simulation*" OR "Mixed Reality" OR "Educational Virtual Reality" OR "Instructional Virtual Reality" OR "Patient-Specific Computational Modeling" OR "Interactive Learning") AND ("Cardiovascular Disease*" OR "Heart Disease*" OR "Heart Disorder*" OR "Cardiac Disease*" OR "Cardiac Disorder*" OR "Heart Defect*") AND ("Pediatric*" OR "Child" OR "Children"))) OR KW ((("Computer Simulation*" OR "Augmented Reality" OR "Patient-Specific Modeling" OR "Virtual Reality" OR "Patient Simulation*" OR "Mixed Reality" OR "Educational Virtual Reality" OR "Instructional Virtual Reality" OR "Patient-Specific Computational Modeling" OR "Interactive Learning") AND ("Cardiovascular Disease*" OR "Heart Disease*" OR "Heart Disorder*" OR "Cardiac Disease*" OR "Cardiac Disorder*" OR "Heart Defect*") AND ("Pediatric*" OR "Child" OR "Children"))) AND (Language: English) AND (Publication Date: up to 2025/09/01)
**ProQuest**	ABSTRACT((("Computer Simulation*" OR "Augmented Reality" OR "Patient-Specific Modeling" OR "Virtual Reality" OR "Patient Simulation*" OR "Mixed Reality" OR "Educational Virtual Reality" OR "Instructional Virtual Reality" OR "Patient-Specific Computational Modeling" OR "Interactive Learning") AND ("Cardiovascular Disease*" OR "Heart Disease*" OR "Heart Disorder*" OR "Cardiac Disease*" OR "Cardiac Disorder*" OR "Heart Defect*") AND ("Pediatric*" OR "Child" OR "Children"))) OR TITLE((("Computer Simulation*" OR "Augmented Reality" OR "Patient-Specific Modeling" OR "Virtual Reality" OR "Patient Simulation*" OR "Mixed Reality" OR "Educational Virtual Reality" OR "Instructional Virtual Reality" OR "Patient-Specific Computational Modeling" OR "Interactive Learning") AND ("Cardiovascular Disease*" OR "Heart Disease*" OR "Heart Disorder*" OR "Cardiac Disease*" OR "Cardiac Disorder*" OR "Heart Defect*") AND ("Pediatric*" OR "Child" OR "Children"))) OR IF((("Computer Simulation*" OR "Augmented Reality" OR "Patient-Specific Modeling" OR "Virtual Reality" OR "Patient Simulation*" OR "Mixed Reality" OR "Educational Virtual Reality" OR "Instructional Virtual Reality" OR "Patient-Specific Computational Modeling" OR "Interactive Learning") AND ("OR "Heart Disease*" OR "Heart Disorder*" OR "Cardiac Disease*" OR "Cardiac Disorder*" OR "Heart Defect*") AND ("Pediatric*" OR "Child" OR "Children"))) AND (Language: English) AND (Date: up to 2025/09/01)
**Google Scholar**	(("Computer-based Simulation*" OR "Patient-Specific Modeling") AND ("Cardiovascular Disease*") AND ("Pediatric*")) with custom date range: 2000–2025 and language: English
