# Supplementary material for: Computer-Based Simulation Technologies in Pediatric Cardiovascular Diseases: A Systematic Review of Applications and Outcomes
Source: Healthcare (Basel). 2026 Sep 19;14(18):3086. doi: 10.3390/healthcare14183086 (PMC13606252; doi:10.3390/healthcare14183086)
Supplement: Supplementary file 1 [file healthcare-14-03086-s001.zip › Supplementary Table S3 - Critical appraisal and Risk of bias.pdf]

Critical Appraisal

Mixed Methods Appraisal Tool (MMAT)

	Screening Questions		Quantitative non-randomized					Quantitative Descriptive					Mixed Methods					Qualitative							
Author (year of publication)	Are there clear research questions?	Does the collected data allow to address the research questions?	Are the participants representative of the target population?	Are measurements appropriate regarding both the outcome and intervention (or exposure)?	Are there complete outcome data?	Are the confounders accounted for in the design and analysis?	During the study period, is the intervention administered (or exposure occurred) as intended?	Is the sampling strategy relevant to address the research question?	Is the sample representative of the target population?	Are the measurements appropriate?	Is the risk of nonresponse bias low?	Is statistical analysis appropriate to answer the research question?	Is there an adequate rationale for using a mixed methods design to address the research question?	Are the different components of the study effectively integrated to answer the research question?	Are the outputs of the integration of qualitative and quantitative components adequately interpreted?	Are divergences and inconsistencies between quantitative and qualitative results adequately addressed?	Do the different components of the study adhere to the quality criteria of each tradition of the methods involved?	Is the qualitative approach appropriate to answer the research question?	Are the qualitative data collection methods adequate to address the research question?	Are the findings adequately derived from the data?	. Is the interpretation of results sufficiently substantiated by data?	. Is the interpretation of results sufficiently substantiated by data?	Total Points	Score	Quality
Riesenkampff et al. / 2009 (1)	Y	Y	N/A	N/A	N/A	N/A	N/A	Y	C	Y	C	Y	N/A	N/A	N/A	N/A	N/A	N/A	N/A	N/A	N/A	N/A	5/7	0.71	**Good**
Xue et al. / 2010 (2)	Y	Y	C	Y	Y	C	C	N/A	N/A	N/A	N/A	N/A	N/A	N/A	N/A	N/A	N/A	N/A	N/A	N/A	N/A	N/A	4/7	0.57	**Moderate**
De Zélicourt et al. / 2011 (3)	Y	Y	C	Y	C	C	Y	N/A	N/A	N/A	N/A	N/A	N/A	N/A	N/A	N/A	N/A	N/A	N/A	N/A	N/A	N/A	4/7	0.57	**Moderate**
Huang et al. / 2021 (4)	Y	Y	N/A	N/A	N/A	N/A	N/A	N/A	N/A	N/A	N/A	N/A	N/A	N/A	N/A	N/A	N/A	Y	Y	Y	Y	Y	7/7	1	**Excellent**
Rao et al. / 2015 (5)	Y	Y	C	Y	Y	C	Y	N/A	N/A	N/A	N/A	N/A	N/A	N/A	N/A	N/A	N/A	N/A	N/A	N/A	N/A	N/A	5/7	0.71	**Good**
Pereira et al. / 2016 (6)	Y	Y	C	Y	Y	C	Y	N/A	N/A	N/A	N/A	N/A	N/A	N/A	N/A	N/A	N/A	N/A	N/A	N/A	N/A	N/A	5/7	0.71	**Good**
Olivieri et al. / 2018 (7)	Y	Y	C	Y	Y	Y	Y	N/A	N/A	N/A	N/A	N/A	N/A	N/A	N/A	N/A	N/A	N/A	N/A	N/A	N/A	N/A	6/7	0.85	**Excellent**
Ong et al. / 2018 (8)	Y	Y	C	Y	Y	C	Y	N/A	N/A	N/A	N/A	N/A	N/A	N/A	N/A	N/A	N/A	N/A	N/A	N/A	N/A	N/A	5/7	0.71	**Good**
Brun et al. / 2019 (9)	Y	Y	C	Y	Y	C	Y	N/A	N/A	N/A	N/A	N/A	N/A	N/A	N/A	N/A	N/A	N/A	N/A	N/A	N/A	N/A	5/7	0.71	**Good**
Capellini et al. / 2020 (10)	Y	Y	C	Y	Y	C	C	N/A	N/A	N/A	N/A	N/A	N/A	N/A	N/A	N/A	N/A	N/A	N/A	N/A	N/A	N/A	4/7	0.57	**Moderate**
Priya et al./ 2020 (11)	Y	Y	N/A	N/A	N/A	N/A	N/A	Y	C	Y	C	C	N/A	N/A	N/A	N/A	N/A	N/A	N/A	N/A	N/A	N/A	4/7	0.57	**Moderate**
Kang et al. / 2020 (12)	Y	Y	C	Y	Y	C	C	N/A	N/A	N/A	N/A	N/A	N/A	N/A	N/A	N/A	N/A	N/A	N/A	N/A	N/A	N/A	4/7	0.57	**Moderate**
Cen et al. / 2021 (13)	Y	Y	C	Y	Y	C	Y	N/A	N/A	N/A	N/A	N/A	N/A	N/A	N/A	N/A	N/A	N/A	N/A	N/A	N/A	N/A	5/7	0.71	**Good**
Gehrsitz et al./ 2021 (14)	Y	Y	N/A	N/A	N/A	N/A	N/A	Y	C	Y	C	Y	N/A	N/A	N/A	N/A	N/A	N/A	N/A	N/A	N/A	N/A	5/7	0.71	**Good**
Milano et al. / 2021 (15)	Y	Y	N/A	N/A	N/A	N/A	N/A	N/A	N/A	N/A	N/A	N/A	Y	Y	Y	Y	Y	N/A	N/A	N/A	N/A	N/A	7/7	1	**Excellent**
Pushparajah et al. / 2021 (16)	Y	Y	N/A	N/A	N/A	N/A	N/A	Y	C	Y	C	Y	**N/A**	N/A	N/A	N/A	N/A	N/A	N/A	N/A	N/A	N/A	5/7	0.71	**Good**
Raimondi et al. / 2021 (17)	Y	Y	N/A	N/A	N/A	N/A	N/A	Y	C	Y	C	N	N/A	N/A	N/A	N/A	N/A	N/A	N/A	N/A	N/A	N/A	4/7	0.57	**Moderate**
van de Woestijne et al. / 2021 (18)	Y	Y	N/A	N/A	N/A	N/A	N/A	Y	C	Y	C	C	N/A	N/A	N/A	N/A	N/A	N/A	N/A	N/A	N/A	N/A	4/7	0.57	**Moderate**
Vigil et al./ 2021 (19)	Y	Y	C	Y	Y	Y	Y	N/A	N/A	N/A	N/A	N/A	N/A	N/A	N/A	N/A	N/A	N/A	N/A	N/A	N/A	N/A	6/7	0.85	**Excellent**
Ye et al. / 2021 (20)	Y	Y	N/A	N/A	N/A	N/A	N/A	Y	C	Y	C	Y	N/A	N/A	N/A	N/A	N/A	N/A	N/A	N/A	N/A	N/A	5/7	0.71	**Good**
Ghosh et al. / 2022(21)	Y	Y	N/A	N/A	N/A	N/A	N/A	Y	C	Y	C	Y	N/A	N/A	N/A	N/A	N/A	N/A	N/A	N/A	N/A	N/A	5/7	0.71	**Good**
Vegulla et al. / 2022 (22)	Y	Y	N/A	N/A	N/A	N/A	N/A	Y	C	Y	C	Y	N/A	N/A	N/A	N/A	N/A	N/A	N/A	N/A	N/A	N/A	5/7	0.71	**Good**
Stepanenko et al./ 2023 (23)	Y	Y	Y	Y	C	Y	Y	N/A	N/A	N/A	N/A	N/A	N/A	N/A	N/A	N/A	N/A	N/A	N/A	N/A	N/A	N/A	6/7	0.85	**Excellent**
Italiano et al./ 2023 (24)	Y	Y	N/A	N/A	N/A	N/A	N/A	Y	N	Y	C	C	N/A	N/A	N/A	N/A	N/A	N/A	N/A	N/A	N/A	N/A	4/7	0.57	**Moderate**
Peek et al./ 2023 (25)	Y	Y	N/A	N/A	N/A	N/A	N/A	Y	C	Y	C	N	N/A	N/A	N/A	N/A	N/A	N/A	N/A	N/A	N/A	N/A	4/7	0.57	**Moderate**
Miyagi et al./ 2023 (26)	Y	Y	N/A	N/A	N/A	N/A	N/A	Y	N	Y	C	Y	N/A	N/A	N/A	N/A	N/A	N/A	N/A	N/A	N/A	N/A	5/7	0.71	**Good**
Bertelli et al./ 2023 (27)	Y	Y	N/A	N/A	N/A	N/A	N/A	Y	C	Y	C	Y	N/A	N/A	N/A	N/A	N/A	N/A	N/A	N/A	N/A	N/A	5/7	0.71	**Good**
Awori et al./ 2023 (28)	Y	Y	N/A	N/A	N/A	N/A	N/A	Y	C	Y	C	Y	N/A	N/A	N/A	N/A	N/A	N/A	N/A	N/A	N/A	N/A	5/7	0.71	**Good**
Lim et al./ 2023 (29)	Y	Y	Y	Y	Y	C	Y	N/A	N/A	N/A	N/A	N/A	N/A	N/A	N/A	N/A	N/A	N/A	N/A	N/A	N/A	N/A	6/7	0.85	**Excellent**

Salavitabar et al./ 2024 (30)	Y	Y	Y	Y	Y	C	Y	N/A	N/A	N/A	N/A	N/A	N/A	N/A	N/A	N/A	N/A	N/A	N/A	N/A	N/A	N/A	6/7	0.85	**Excellent**
Mehta et al./ 2024 (31)	Y	Y	N/A	N/A	N/A	N/A	N/A	N/A	N/A	N/A	N/A	N/A	Y	Y	Y	C	C	N/A	N/A	N/A	N/A	N/A	5/7	0.71	**Good**
Blonsky et al. / 2025 (32)	Y	Y	Y	Y	Y	C	Y	N/A	N/A	N/A	N/A	N/A	N/A	N/A	N/A	N/A	N/A	N/A	N/A	N/A	N/A	N/A	6/7	0.85	**Excellent**
Tiwari et al. / 2025 (33)	Y	Y	N/A	N/A	N/A	N/A	N/A	Y	C	Y	Y	Y	N/A	N/A	N/A	N/A	N/A	N/A	N/A	N/A	N/A	N/A	6/7	0.85	**Excellent**
Schlegel et al. /2025 (34)	Y	Y	N/A	N/A	N/A	N/A	N/A	Y	Y	C	Y	N	N/A	N/A	N/A	N/A	N/A	N/A	N/A	N/A	N/A	N/A	5/7	0.71	**Good**
Ponzoni et al. / 2025 (35)	Y	Y	N/A	N/A	N/A	N/A	N/A	Y	C	Y	C	C	N/A	N/A	N/A	N/A	N/A	N/A	N/A	N/A	N/A	N/A	4/7	0.57	**Moderate**

Abbreviations: C= Can't tell; Y= Yes; N= No; N/A = Not Applicable; Quality Items: Poor (≤ 0.40), Moderate (0.41 to 0.59), Good (0.60 to 0.79), or Excellent (≥ 0.80)

JBI Checklist for Case Reports

Author (year of publication)	Q1	Q2	Q3	Q4	Q5	Q6	Q7	Q8	Total Points	Score	Quality
Jolley et al. / 2024 (36)	Y	Y	Y	Y	Y	Y	Y	Y	8/8	1	High

Abbreviations: Y= Yes; N= No; U=Unclear ; N/A = Not Applicable; Q1:Were patient's demographic characteristics clearly described?, Q2:Was the patient's history clearly described and presented as a timeline?, Q3: Was the current clinical condition of the patient on presentation clearly described?, Q4: Were diagnostic tests or assessment methods and the results clearly described? , Q5: Was the intervention(s) or treatment procedure(s) clearly described?, Q6: Was the post-intervention clinical condition clearly described?, Q7: Were adverse events (harms) or unanticipated events identified and described?, Q8: Does the case report provide takeaway lessons?;; Quality Items: Low (≤ 0.50), Moderate (0.51 to 0.85), High (≥ 0.85)

Risk of Bias

CASP Checklist for Qualitative Study Risk of Bias

Author (year of publication)	Item 1	Item 2	Item 3	Item 4	Item 5	Item 6	Item 7	Item 8	Item 9	Item 10	Score	Overall Appraisal
Huang et al. / 2021 (4)	Y	Y	Y	Can't Tell	N	Can't Tell	Can't Tell	Can't Tell	Y	Y	5	Medium

Abbreviations: Y = Yes; N = No; Item 1 = Clear statement of aim; Item 2 = Suitable qualitative methodology; Item 3 = Appropriate research design; Item 4 = Proper recruiting strategy; Item 5 = Adequacy in data collection; Item 6 = Adequate relationship between researcher and participants; Item 7 = Ethical considerations; Item 8 = Rigor in data analysis; Item 9 = Clear statement of findings; Item 10 = Overall value of research (usefulness of results locally).

Quantitative Risk of Bias - ROBINS-I

	Bias Domain							
Author (year of publication)	Bias due to confounding	Bias in selection of participants into the study	Bias in classification of interventions	Bias due to deviations from intended interventions	Bias due to missing data	Bias in measurement of outcomes	Bias in selection of the reported result	Overall bias
Riesenkampff et al. / 2009 (1)	Moderate	Moderate	Low	Low	Low	Moderate	Moderate	Moderate
Xue et al. / 2010 (2)	Moderate	Moderate	Moderate	Low	Moderate	Moderate	Moderate	Moderate
De Zélicourt et al. / 2011 (3)	Low	Low	Low	Low	Low	Moderate	Low	Low
Rao et al. / 2015 (5)	Low	Moderate	Low	Moderate	Moderate	Low	Moderate	Moderate
Pereira et al. / 2016 (6)	Moderate	Moderate	Low	Low	Serious	Moderate	Low	Moderate
Olivieri et al. / 2018 (7)	Low	Moderate	Low	Moderate	Serious	Low	Moderate	Moderate
Ong et al. / 2018 (8)	Low	Low	Low	Low	Low	Low	Low	Low
Brun et al. / 2019 (9)	Low	Moderate	Moderate	Low	Low	Low	Moderate	Moderate
Capellini et al. / 2020 (10)	Moderate	Moderate	Low	Low	Low	Moderate	Moderate	Moderate

Author (year of publication)	Bias due to confounding	Bias in selection of participants into the study	Bias in classification of interventions	Bias due to deviations from intended interventions	Bias due to missing data	Bias in measurement of outcomes	Bias in selection of the reported result	Overall bias
Priya et al./ 2020 [11]	Moderate	Low	Low	Low	Moderate	Moderate	Moderate	Moderate
Kang et al. / 2020 (12)	Low	Moderate	Low	Moderate	Low	Moderate	Low	Moderate
Cen et al. / 2021 (13)	Low	Moderate	Moderate	Moderate	Low	Moderate	Low	Moderate
Gehrsitz et al./ 2021 (14)	Low	Low	Low	Low	Low	Low	Low	Low
Milano et al. / 2021 (15)	Moderate	Low	Low	Low	Moderate	Moderate	Moderate	Moderate
Pushparajah et al. / 2021 (16)	Low	Low	Low	Low	Moderate	Low	Low	Low
Raimondi et al. / 2021 (17)	Low	Low	Low	Low	Low	Low	Low	Low
van de Woestijne et al. / 2021 (18)	Low	Low	Moderate	Low	Moderate	Moderate	Moderate	Moderate
Vigil et al./ 2021 (19)	Moderate	Moderate	Low	Low	Low	Low	Moderate	Moderate
Ye et al. / 2021 (20)	Low	Low	Low	Low	Low	Moderate	Low	Low
Ghosh et al. / 2022(21)	Low	Low	Low	Low	Low	Low	Low	Low
Vegulla et al. / 2022 (22)	Low	Low	Low	Low	Low	Low	Low	Low
Italiano et al./ 2023 (24)	Serious	Moderate	Low	Low	Low	Low	Moderate	Moderate
Stepanenko et al./ 2023 (23)	Moderate	Moderate	Low	Low	Moderate	Moderate	Low	Moderate
Peek et al./ 2023 (25)	Moderate	Moderate	Low	Low	Low	Low	Moderate	Low
Miyagi et al./ 2023 (26)	Low	Moderate	Low	Low	Low	Moderate	Low	Low
Lim et al./ 2023 (29)	Moderate	Moderate	Low	Low	Moderate	Low	Low	Moderate
Bertelli et al./ 2023 (27)	Low	Moderate	Low	Low	Low	Moderate	Low	Low
Awori et al./ 2023 (28)	Moderate	Low	Low	Low	Low	Moderate	Low	Low
Salavitabar et al./ 2024 (30)	Moderate	Moderate	Low	Low	Low	Moderate	Low	Moderate
Jolley et al. / 2024 (36)	Serious	Serious	Low	Low	Low	Low	Low	Moderate
Mehta et al./ 2024 (31)	Low	Moderate	Low	Low	Low	Moderate	Moderate	Moderate
Tiwari et al. / 2025 (33)	Serious	Low	Low	Low	Low	Moderate	Moderate	Moderate
Schlegel et al. /2025 (34)	Low	Moderate	N/A	N/A	Moderate	Low	Low	Moderate
Ponzoni et al. / 2025 (35)	Serious	Serious	Low	Low	Low	Moderate	Moderate	Serious
Blonsky et al. / 2025 (32)	Moderate	Low	Low	Low	Low	Low	Low	Low

Abbreviations: Low = Low risk of bias; Moderate = Moderate risk of bias; Serious = Serious risk of bias; Critical = Critical risk of bias; Null = No Information; N/A: Not Applicable.

References

1. Riesenkampff E, Rietdorf U, Wolf I, Schnackenburg B, Ewert P, Huebler M, et al. The practical clinical value of three-dimensional models of complex congenitally malformed hearts. J Thorac Cardiovasc Surg. 2009;138(3):571-80.
2. Xue H, Sun K, Yu J, Chen B, Chen G, Hong W, et al. Three-dimensional echocardiographic virtual endoscopy for the diagnosis of congenital heart disease in children. The international journal of cardiovascular imaging. 2010;26(8):851-9.
3. De Zélicourt DA, Haggerty CM, Sundareswaran KS, Whited BS, Rossignac JR, Kanter KR, et al. Individualized computer-based surgical planning to address pulmonary arteriovenous malformations in patients with a single ventricle with an interrupted inferior vena cava and azygous continuation. Journal of Thoracic and Cardiovascular Surgery. 2011;141(5):1170-7.
4. Huang J, Plasencia JD, Bardo DME, Huber NC, Ellsworth EG, Zangwill SD, Bryan C, editors. Phoenix Virtual Heart: A Hybrid VR-Desktop Visualization System for Cardiac Surgery Planning and Education. 2021 IEEE Workshop on Visual Analytics in Healthcare (VAHC); 2021 24-25 Oct. 2021.
5. Rao AS, Menon PG. Presurgical planning using image-based in silico anatomical and functional characterization of Tetralogy of Fallot with associated anomalies. Interact Cardiovasc Thorac Surg. 2015;20(2):149-56.
6. Pereira D, Gomes P, Faria S, Cruz-Correia R, Coimbra M. Teaching cardiopulmonary auscultation in workshops using a virtual patient simulation technology - A pilot study. Proceedings of the Annual International Conference of the IEEE Engineering in Medicine and Biology Society, EMBS. 2016;2016-October:3019-22.
7. Olivieri LJ, Zurakowski D, Ramakrishnan K, Su L, Alfares FA, Irwin MR, et al. Novel, 3D Display of Heart Models in the Postoperative Care Setting Improves CICU Caregiver Confidence. World Journal for Pediatric and Congenital Heart Surgery. 2018;9(2):206-13.
8. Ong CS, Krishnan A, Huang CY, Spevak P, Vricella L, Hibino N, et al. Role of virtual reality in congenital heart disease. Congenital heart disease. 2018;13(3):357-61.

9. Brun H, Bugge RAB, Suther LKR, Birkeland S, Kumar R, Pelanis E, Elle OJ. Mixed reality holograms for heart surgery planning: First user experience in congenital heart disease. European Heart Journal Cardiovascular Imaging. 2019;20(8):883-8.
10. Capellini K, Tripicchio P, Vignali E, Gasparotti E, Ali LA, Cantinotti M, et al. 3D Printing and 3D Virtual Models for Surgical and Percutaneous Planning of Congenital Heart Diseases. Int Jnt Conf on Comp Visio Imaging & Comp Graphics Theory & Apps. 2020:281-7.
11. Priya S, Nagpal P. Four-dimensional virtual reality cine cardiac models using free open-source software. Pediatr Radiol. 2020;50:1617-23.
12. Kang S-L, Shkumat N, Dragulescu A, Guerra V, Padfield N, Krutikov K, et al. Mixed-reality view of cardiac specimens: a new approach to understanding complex intracardiac congenital lesions. Pediatric radiology. 2020;50(11):1610-6.
13. Cen J, Liufu R, Wen S, Qiu H, Liu X, Chen X, et al. Three-dimensional printing, virtual reality and mixed reality for pulmonary atresia: early surgical outcomes evaluation. Heart Lung Circ. 2021;30(2):296-302.
14. Gehrsitz P, Rompel O, Schober M, Cesnjevar R, Purbojo A, Uder M, et al. Cinematic Rendering in Mixed-Reality Holograms: A New 3D Preoperative Planning Tool in Pediatric Heart Surgery. Front Cardiovasc Med. 2021;8.
15. Milano EG, Kostolny M, Pajaziti E, Marek J, Regan W, Caputo M, et al. Enhanced 3D visualization for planning biventricular repair of double outlet right ventricle: A pilot study on the advantages of virtual reality. Eur Heart J Digit Health. 2021;2(4):667-75.
16. Pushparajah K, Chu KYK, Deng S, Wheeler G, Gomez A, Kabir S, et al. Virtual reality three-dimensional echocardiographic imaging for planning surgical atrioventricular valve repair. JTCVS Techniques. 2021;7:269-77.
17. Raimondi F, Vida V, Godard C, Bertelli F, Reffo E, Boddaert N, et al. Fast-track virtual reality for cardiac imaging in congenital heart disease. J Card Surg. 2021;36(7):2598-602.
18. van de Woestijne PC, Bakhuis W, Sadeghi AH, Peek JJ, Taverne Y, Bogers A. 3D Virtual Reality Imaging of Major Aortopulmonary Collateral Arteries: A Novel Diagnostic Modality. World J Pediatr Congenit Heart Surg. 2021;12(6):765-72.
19. Vigil C, Lasso A, Ghosh RM, Pinter C, Cianciulli A, Nam HH, et al. Modeling Tool for Rapid Virtual Planning of the Intracardiac Baffle in Double-Outlet Right Ventricle. Ann Thorac Surg. 2021;111(6):2078-83.
20. Ye W, Zhang X, Li T, Luo C, Yang L. Mixed-reality hologram for diagnosis and surgical planning of double outlet of the right ventricle: a pilot study. Clin Radiol. 2021;76(3):237.e1-.e7.
21. Ghosh RM, Jolley MA, Mascio CE, Chen JM, Fuller S, Rome JJ, et al. Clinical 3D modeling to guide pediatric cardiothoracic surgery and intervention using 3D printed anatomic models, computer aided design and virtual reality. J 3D print med. 2022;8(1):11.
22. Vegulla RV, Tandon A, Rathinaswamy J, Cherian KM, Hussain T, Murala JS. Advanced imaging and digitization of preserved heart specimens using virtual reality - A primer. Annals of pediatric cardiology. 2022;15(4):351-7.
23. Stepanenko A, Perez LM, Ferre JC, Ybarra Falcón C, Pérez de la Sota E, San Roman JA, et al. 3D Virtual modelling, 3D printing and extended reality for planning of implant procedure of short-term and long-term mechanical circulatory support devices and heart transplantation. Front Cardiovasc Med. 2023;10:1191705.
24. Italiano EG, Bertelli F, Cao I, Motta R, Di Salvo G, Vida V, Padalino MA. Successful Implantation of HeartMate3 in a Small Child after Multimodality Imaging Pathway to Assess Feasibility. ASAIO Journal. 2024;70(6):e78-e81.
25. Peek JJ, Bakhuis W, Sadeghi AH, Veen KM, Roest AAW, Bruining N, et al. Optimized preoperative planning of double outlet right ventricle patients by 3D printing and virtual reality: A pilot study. Interdisciplinary Cardiovascular and Thoracic Surgery. 2023;37(2).
26. Miyagi C, Ahmad M, Karimov JH, Polakowski AR, Karamlou T, El-Yaman M, et al. Human fitting of pediatric and infant continuous-flow total artificial heart: visual and virtual assessment. Front Cardiovasc Med. 2023;10.
27. Bertelli F, Raimondi F, Godard C, Bergonzoni E, Cattapan C, Gastino E, et al. Fast-Track virtual reality software to facilitate 3-dimensional reconstruction in congenital heart disease. Interdisciplinary Cardiovascular and Thoracic Surgery. 2023;36(6).
28. Awori J, Friedman SD, Howard C, Kronmal R, Buddhe S. Comparative effectiveness of virtual reality (VR) vs 3D printed models of congenital heart disease in resident and nurse practitioner educational experience. J 3D print med. 2023;9(1):2.
29. Lim TR, Wilson HC, Axelrod DM, Werho DK, Handler SS, Yu S, et al. Virtual reality curriculum increases paediatric residents' knowledge of CHDs. Cardiology in the Young. 2023;33(3):410-4.
30. Salavitabar A, Zampi JD, Thomas C, Zanaboni D, Les A, Lowery R, et al. Augmented Reality Visualization of 3D Rotational Angiography in Congenital Heart Disease: A Comparative Study to Standard Computer Visualization. Pediatric Cardiology. 2024;45(8):1759-66.
31. Mehta P, Narayanan R, Karanth H, Yang H, Slesnick TC, Shaw F, Chau DH. Multi-User Mobile Augmented Reality for Cardiovascular Surgical Planning. 2024 IEEE Visualization and Visual Analytics (VIS).201-5.
32. Blonsky SE, Henning RE, Masterson EM, Axelrod DM, Handler SS, Yu S, Owens ST. Virtual Reality Curriculum Increases Pediatric Clerkship Students' Knowledge of Congenital Heart Disease. Pediatric Cardiology. 2025.
33. Tiwari N, Mishra A, Ravi Ramamurthy H, Sharma G, Dhanalakshmi B, Pattanayak S, et al. The role of virtual reality in management of complex congenital heart diseases in comparison to conventional cardiac computed tomography scan. Medical Journal Armed Forces India. 2025.
34. Schlegel PP, Kehrle F, Bugaj TJ, Scholz E, Kovacevic A, Grieshaber P, et al. Augmented Reality in Cardiovascular Education (HoloHeart): Assessment of Students' and Lecturers' Needs and Expectations at Heidelberg University Medical School. Applied Sciences. 2025;15(15):8595.
35. Ponzoni M, Bertelli F, Yoo SJ, Peel B, Seatle H, Honjo O, et al. Mixed reality for preoperative planning and intraoperative assistance of surgical correction of complex congenital heart defects. Journal of Thoracic and Cardiovascular Surgery. 2025;170(2):327-35.e1.
36. Jolley MA, Sulentic A, Amin S, Gupta M, Ching S, Cianciulli A, et al. Introduction of transcatheter edge-to-edge repair in patients with congenital heart disease at a children's hospital. Catheterization and Cardiovascular Interventions. 2024;103(2):326-34.
